# Supplementary material for: Acquired Triazole Resistance Alters Pathogenicity-Associated Features in Candida auris in an Isolate-Dependent Manner
Source: J Fungi (Basel). 2023 Nov 28;9(12):1148. doi: 10.3390/jof9121148 (PMC10744493; doi:10.3390/jof9121148)

## Slide 1
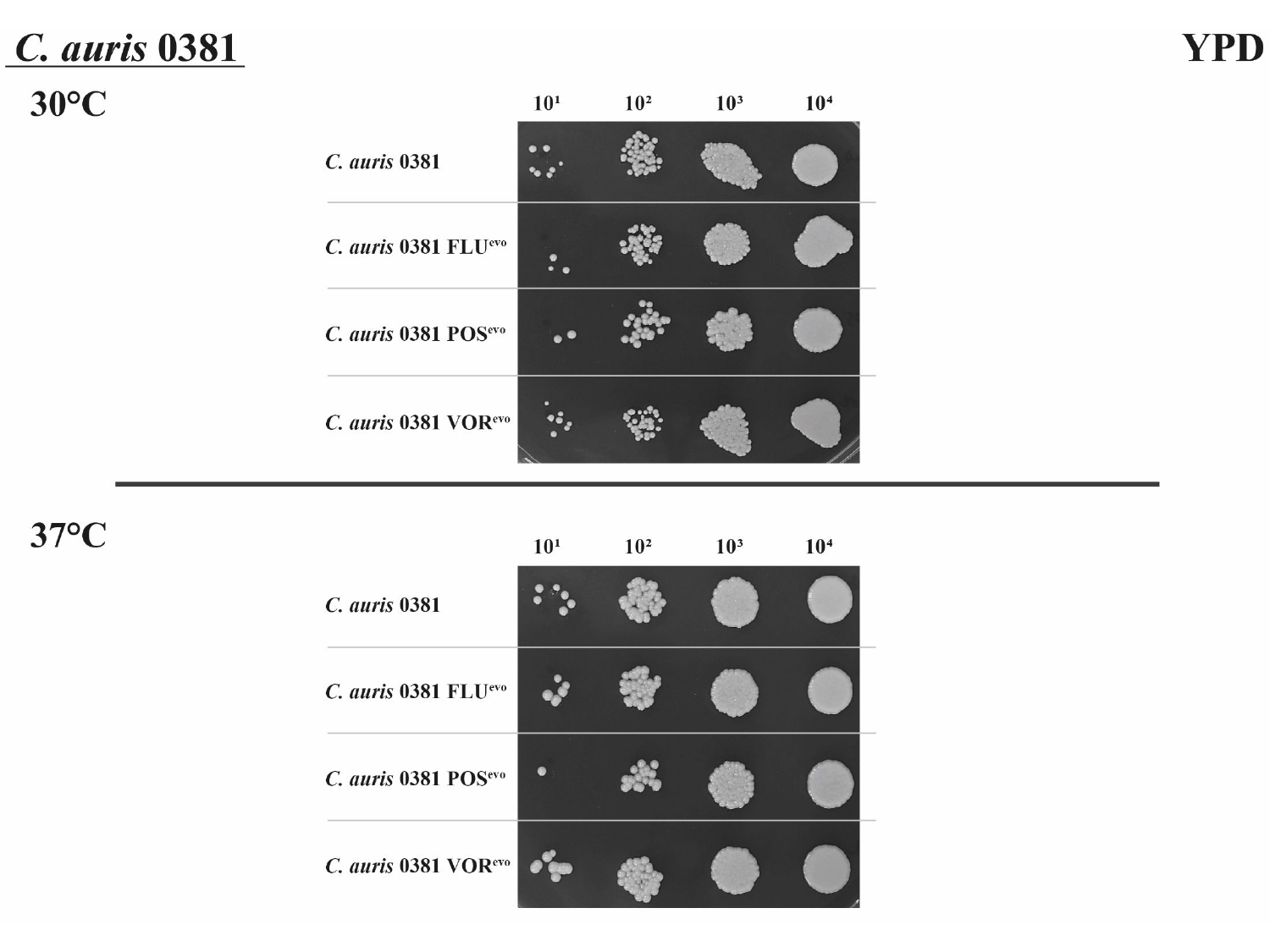

## Slide 2
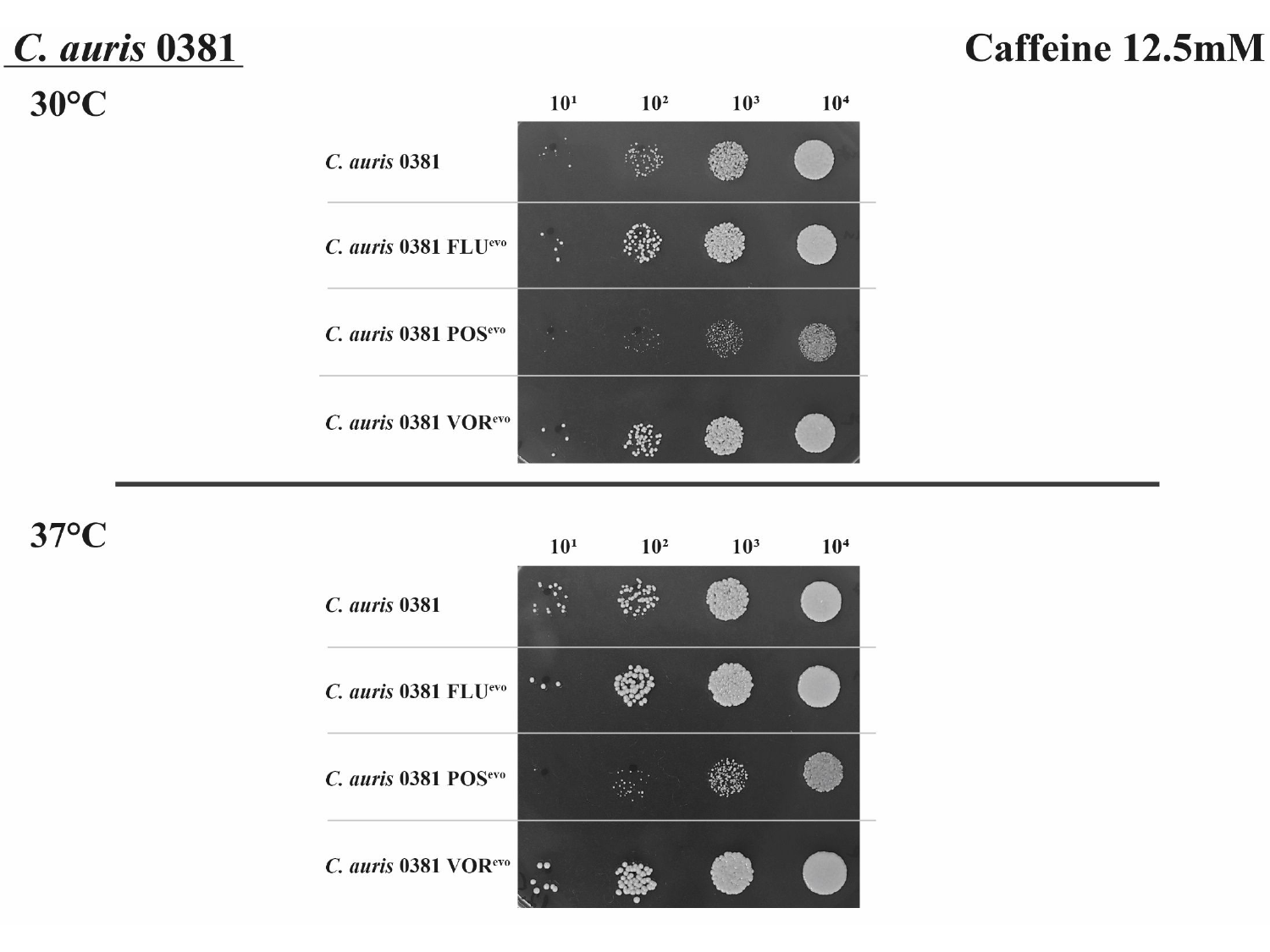

## Slide 3
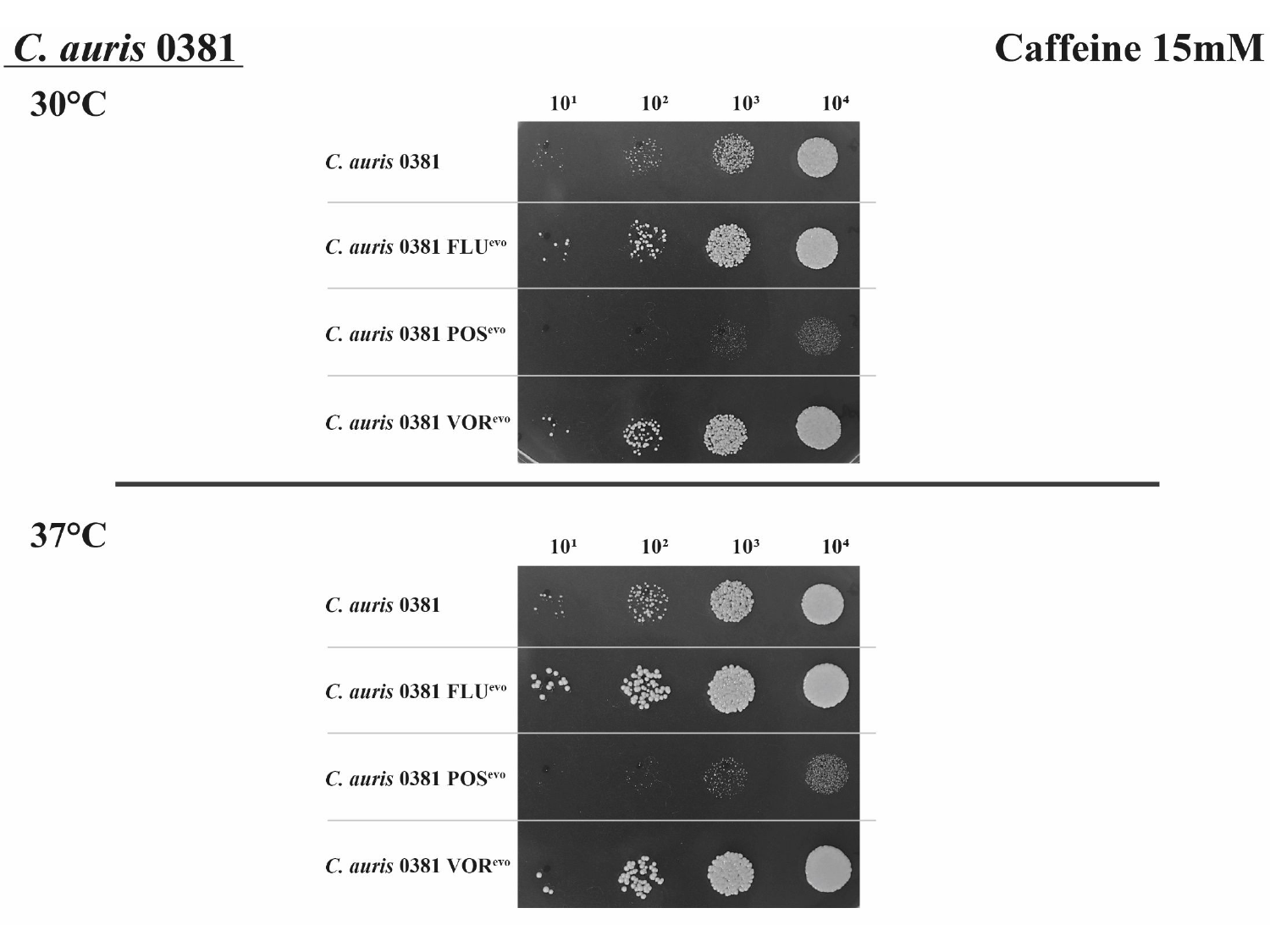

## Slide 4
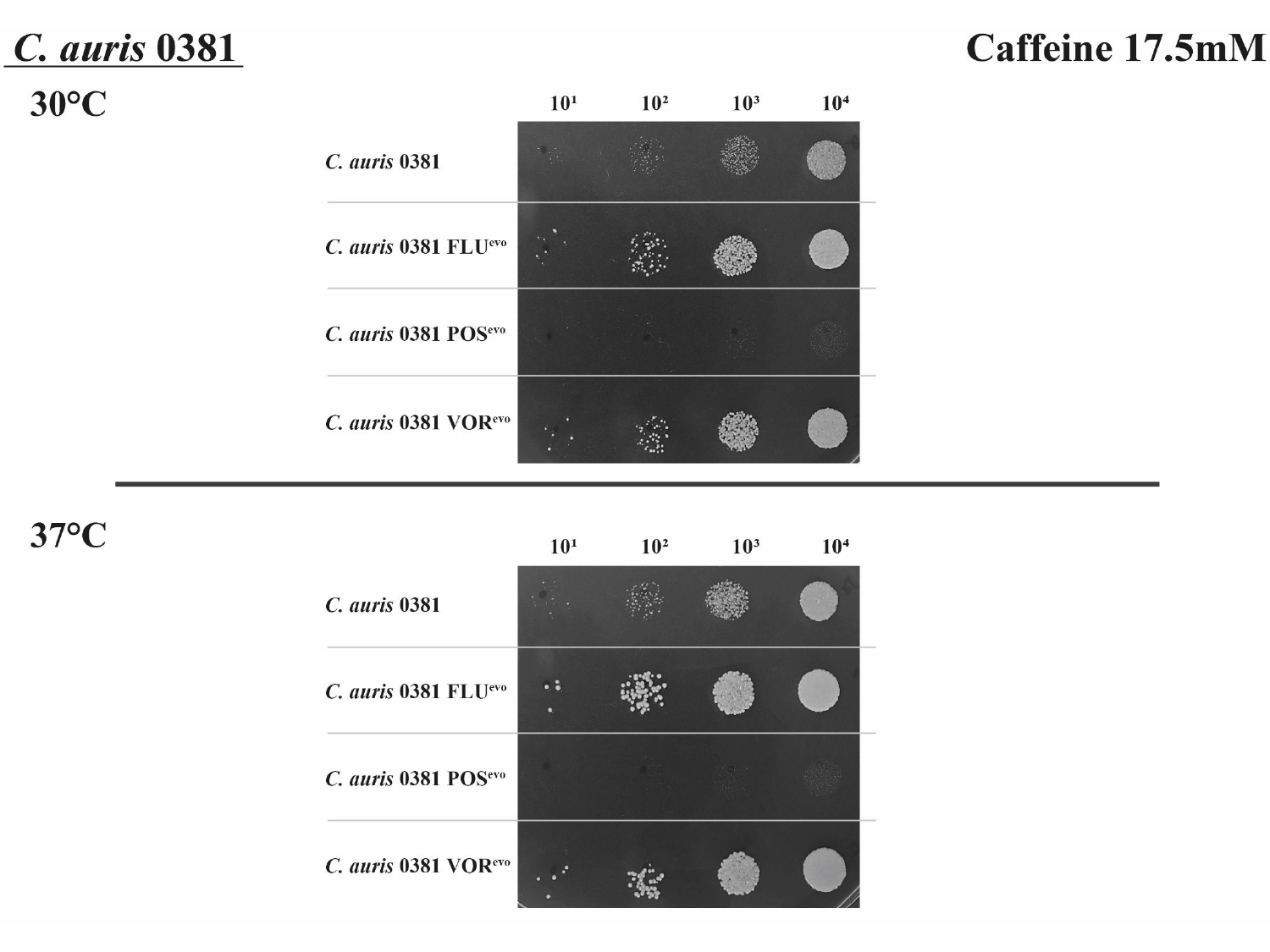

## Slide 5
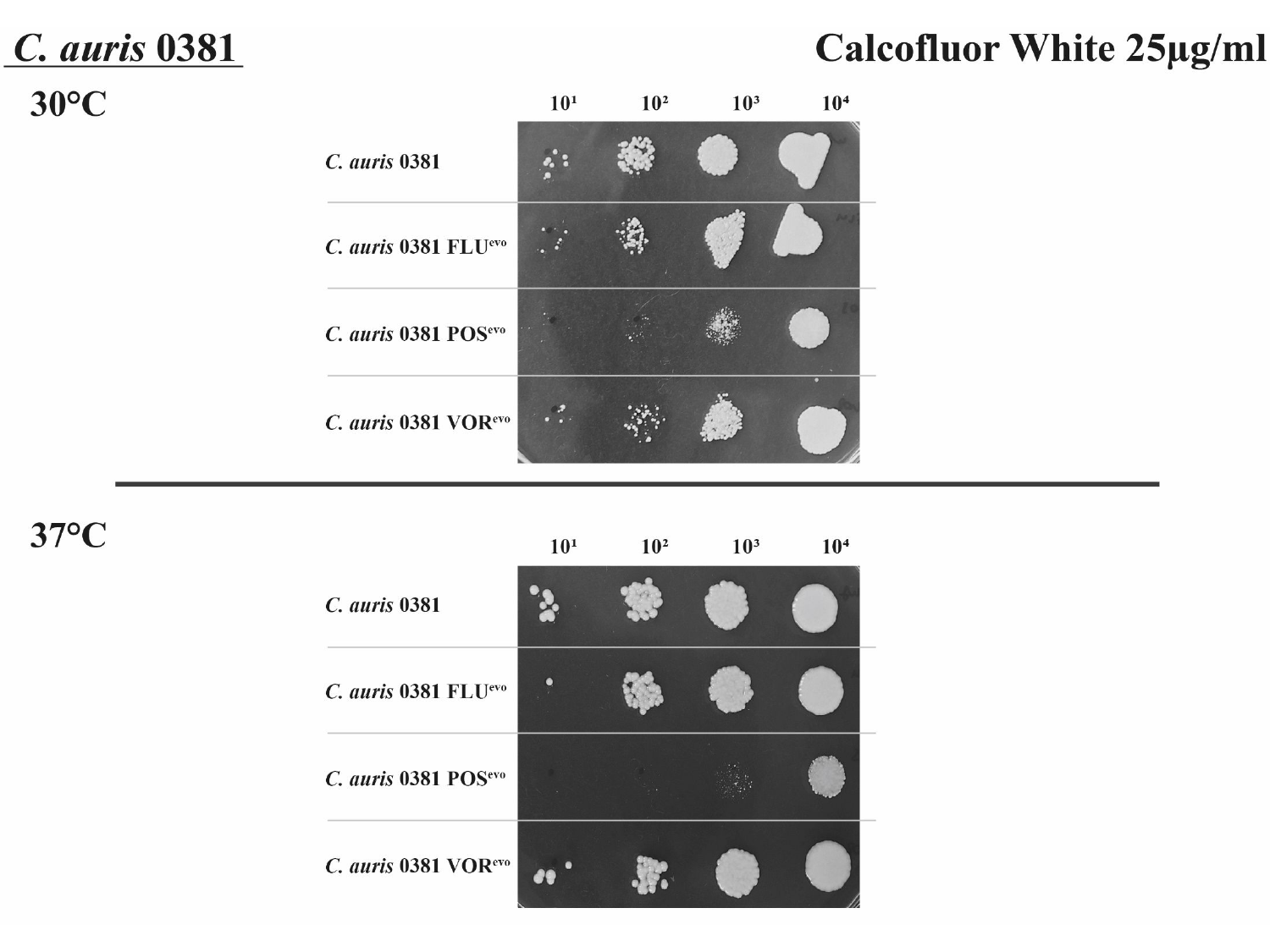

## Slide 6
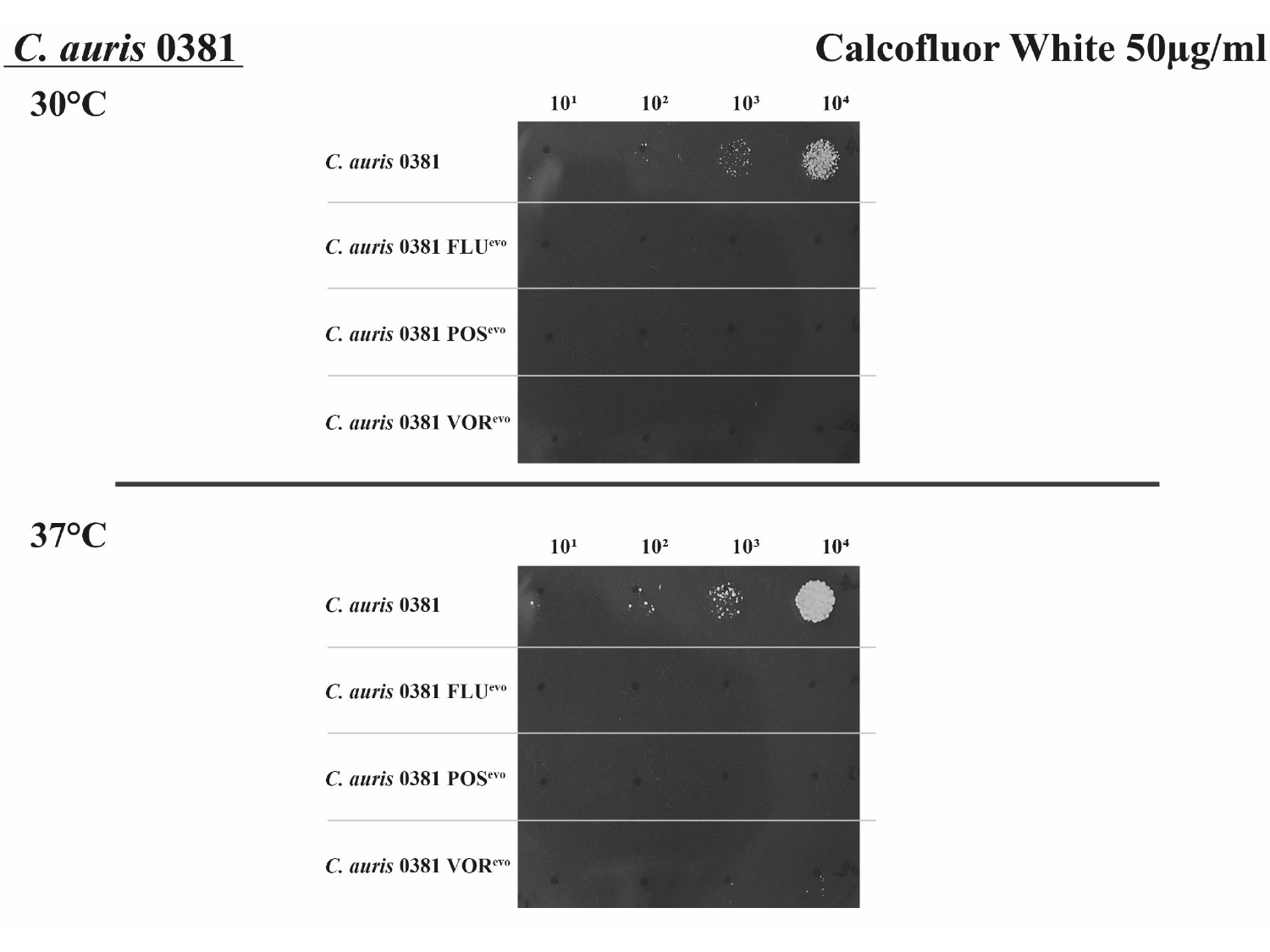

## Slide 7
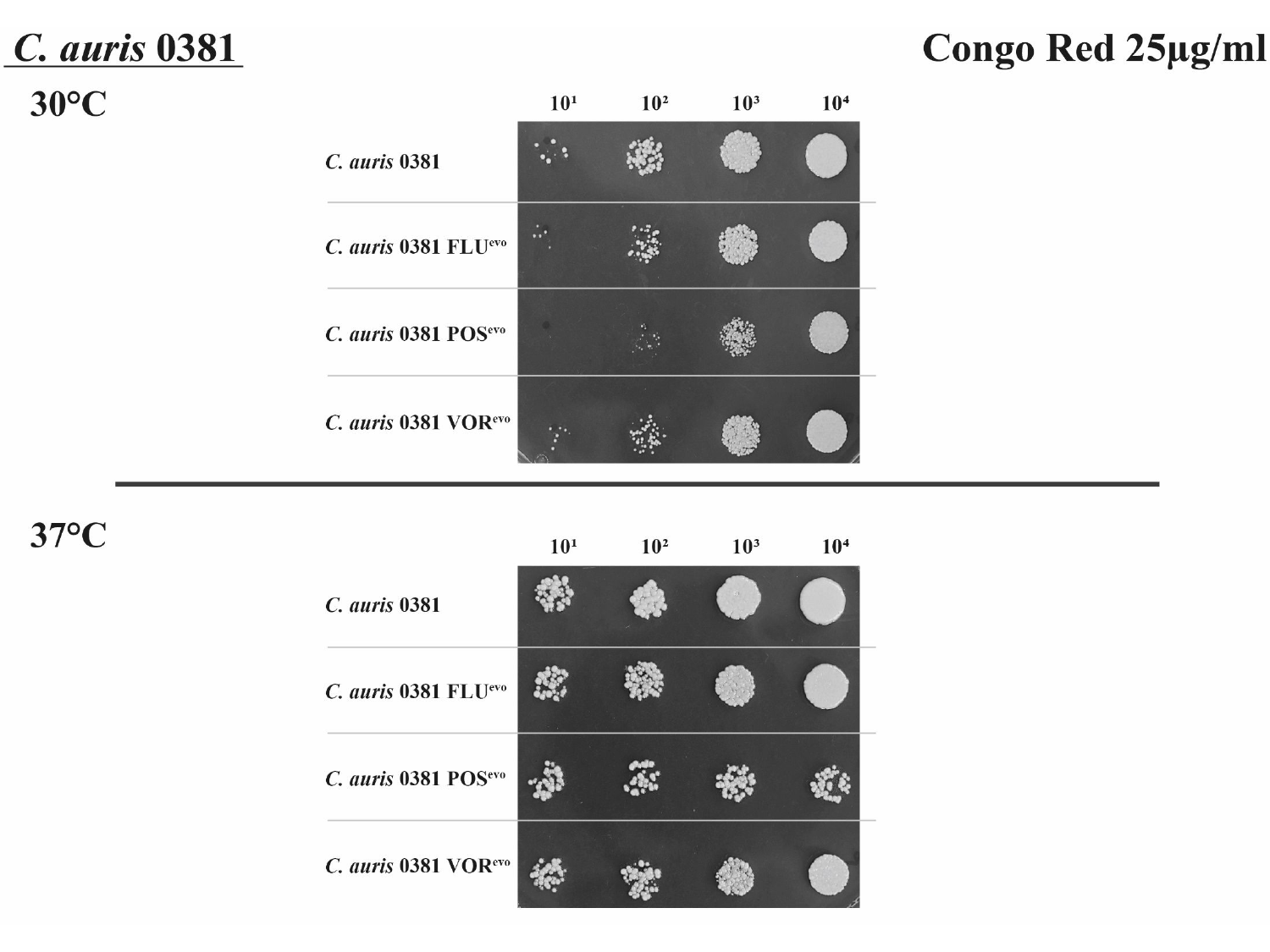

## Slide 8
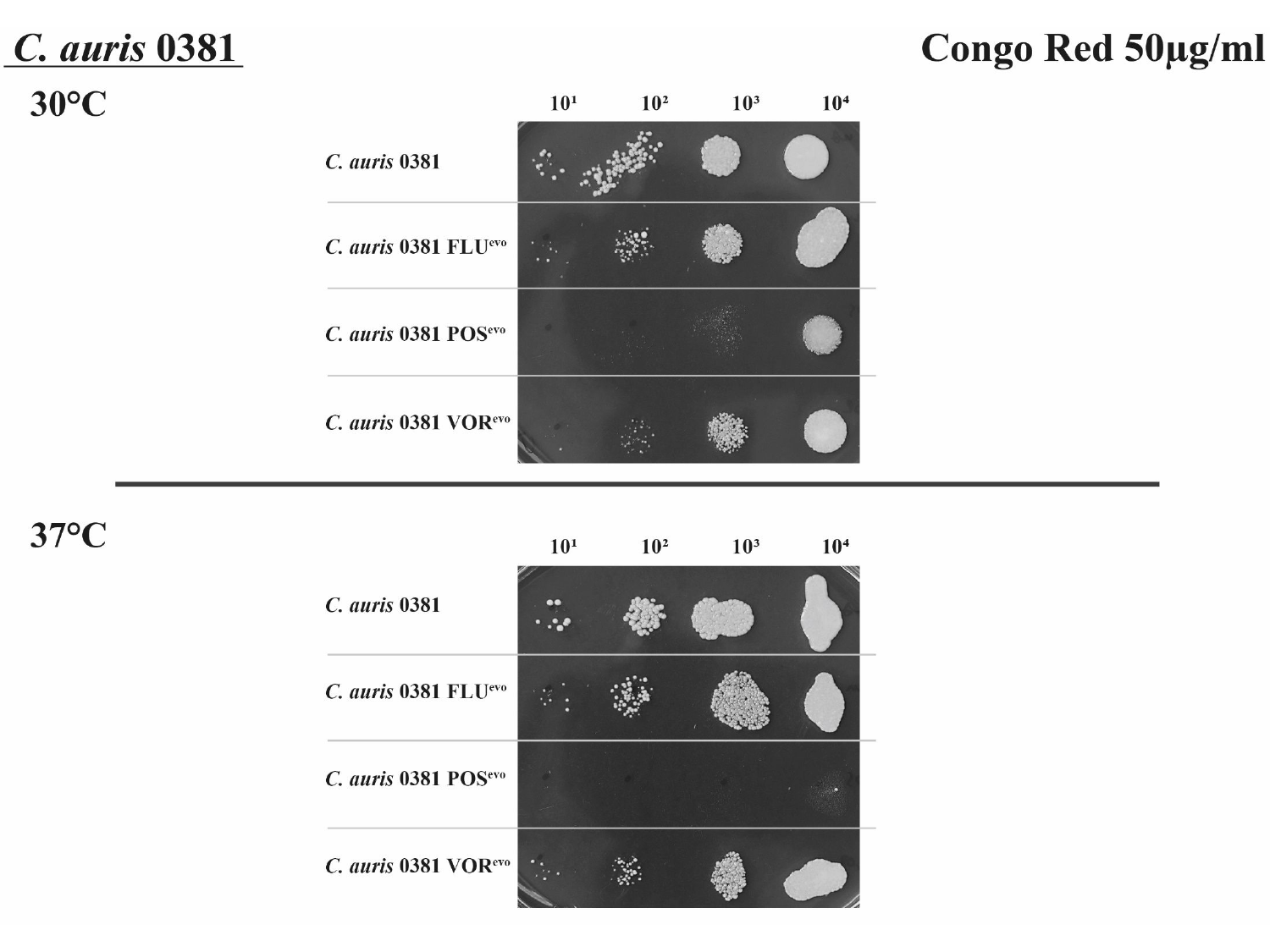

## Slide 9
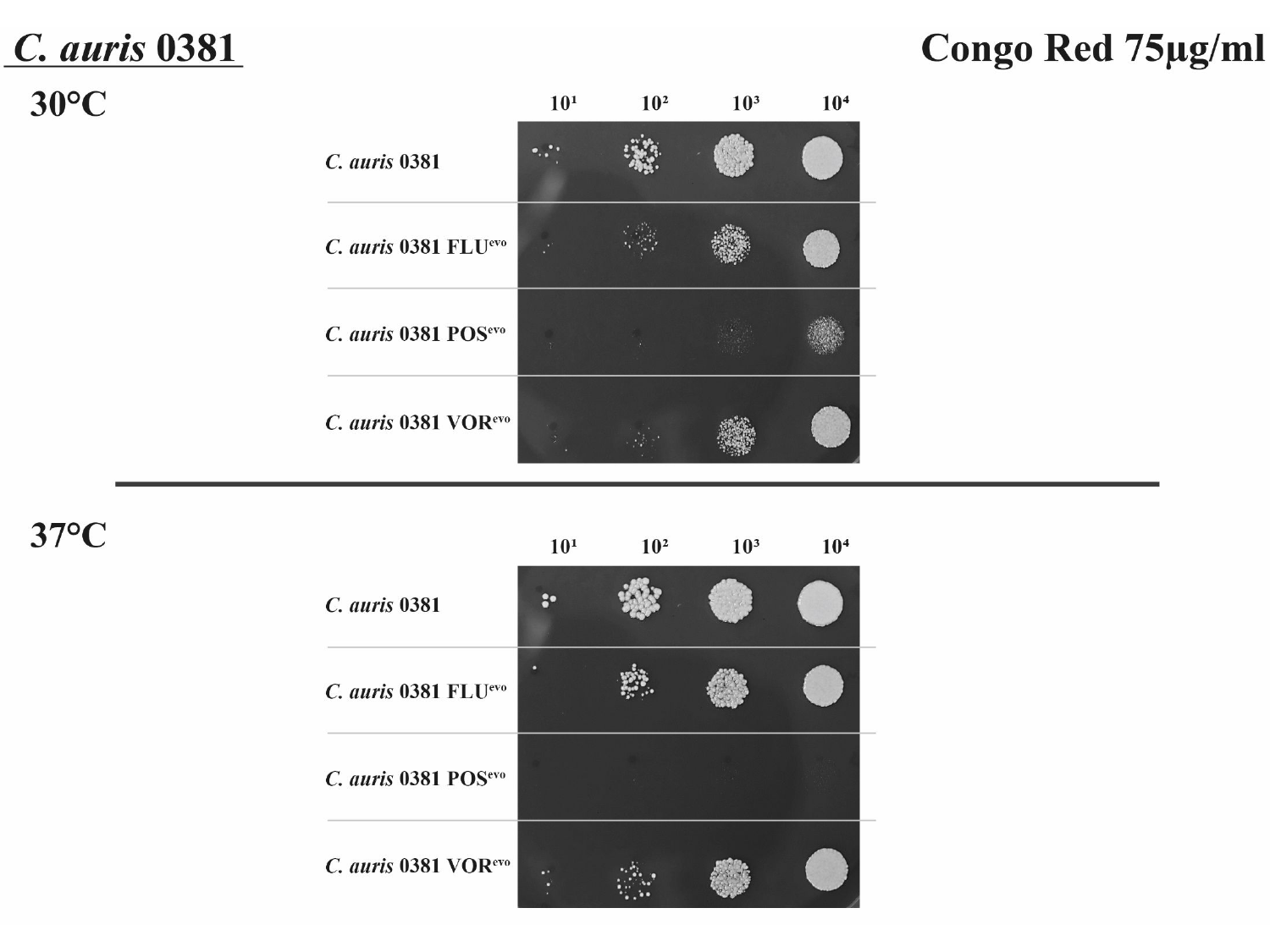

## Slide 10
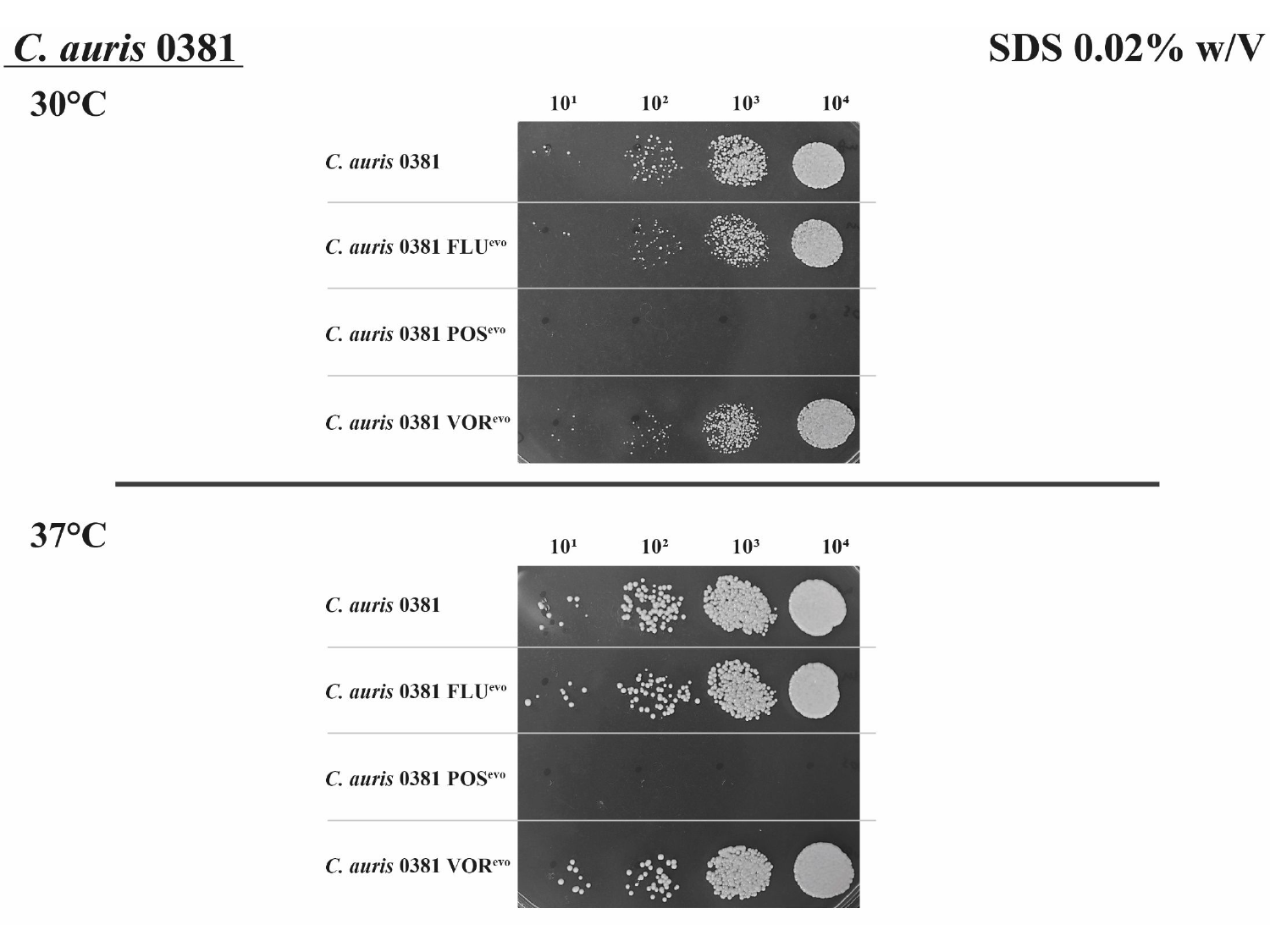

## Slide 11
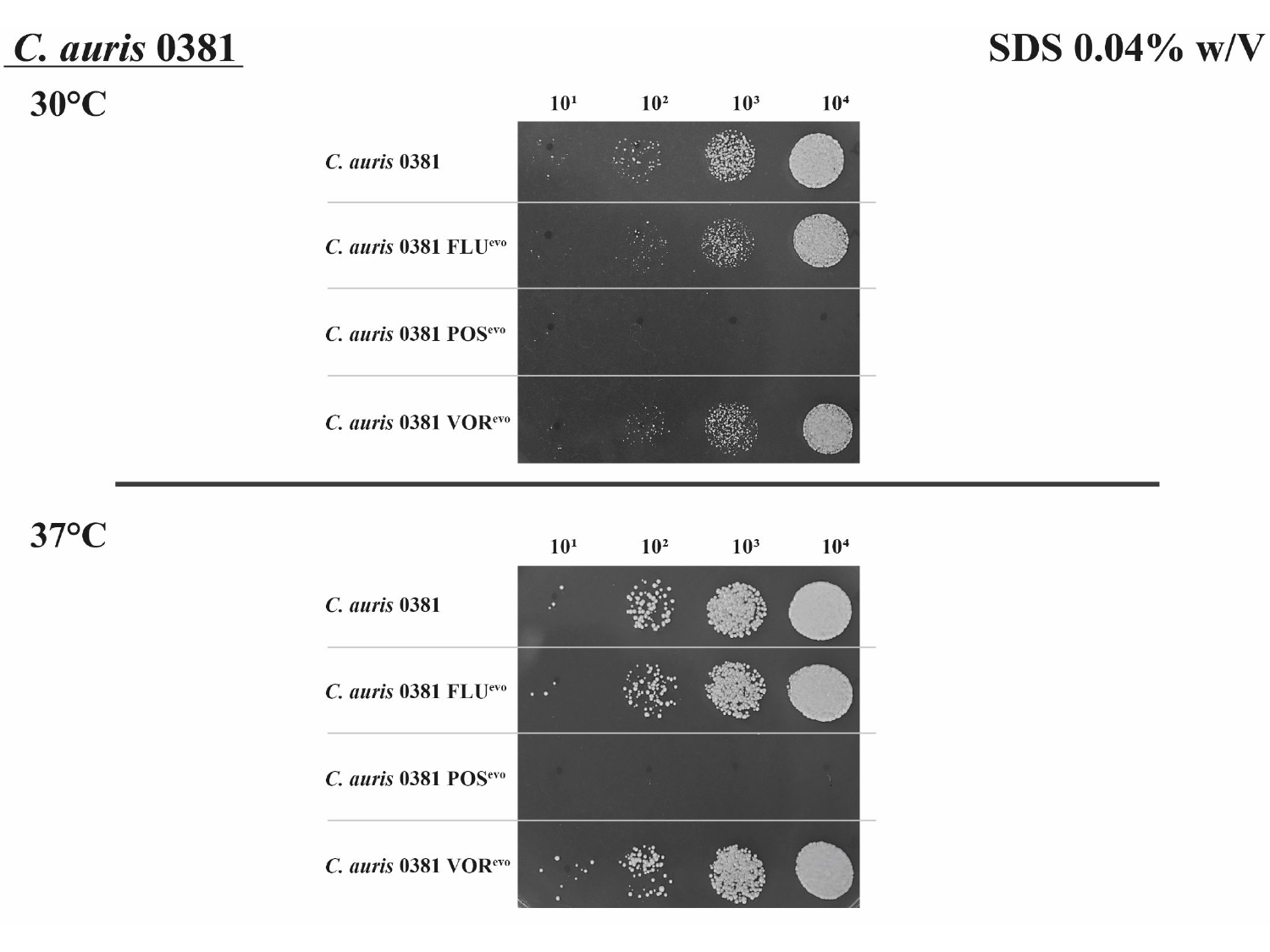

## Slide 12
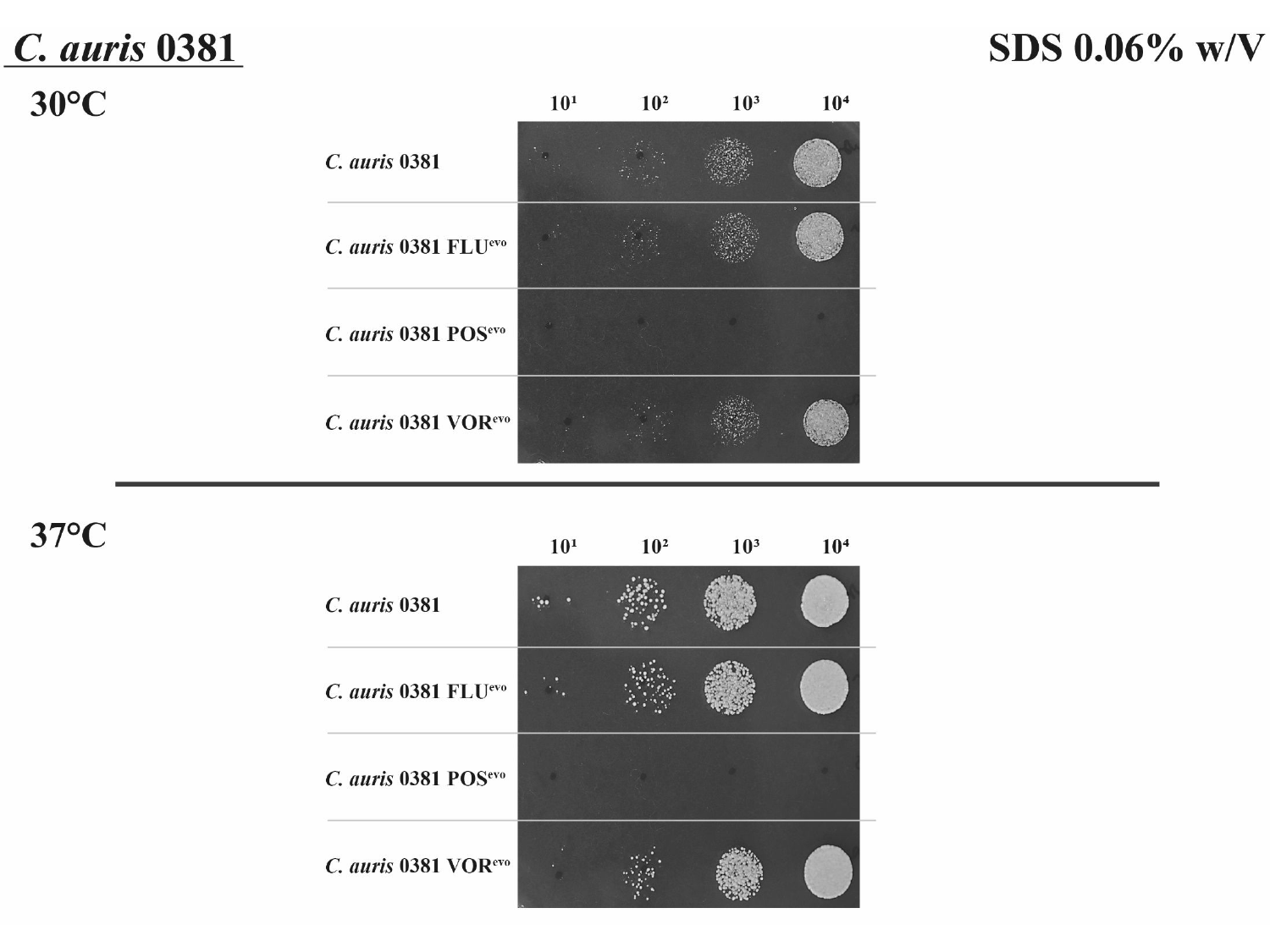

## Slide 13
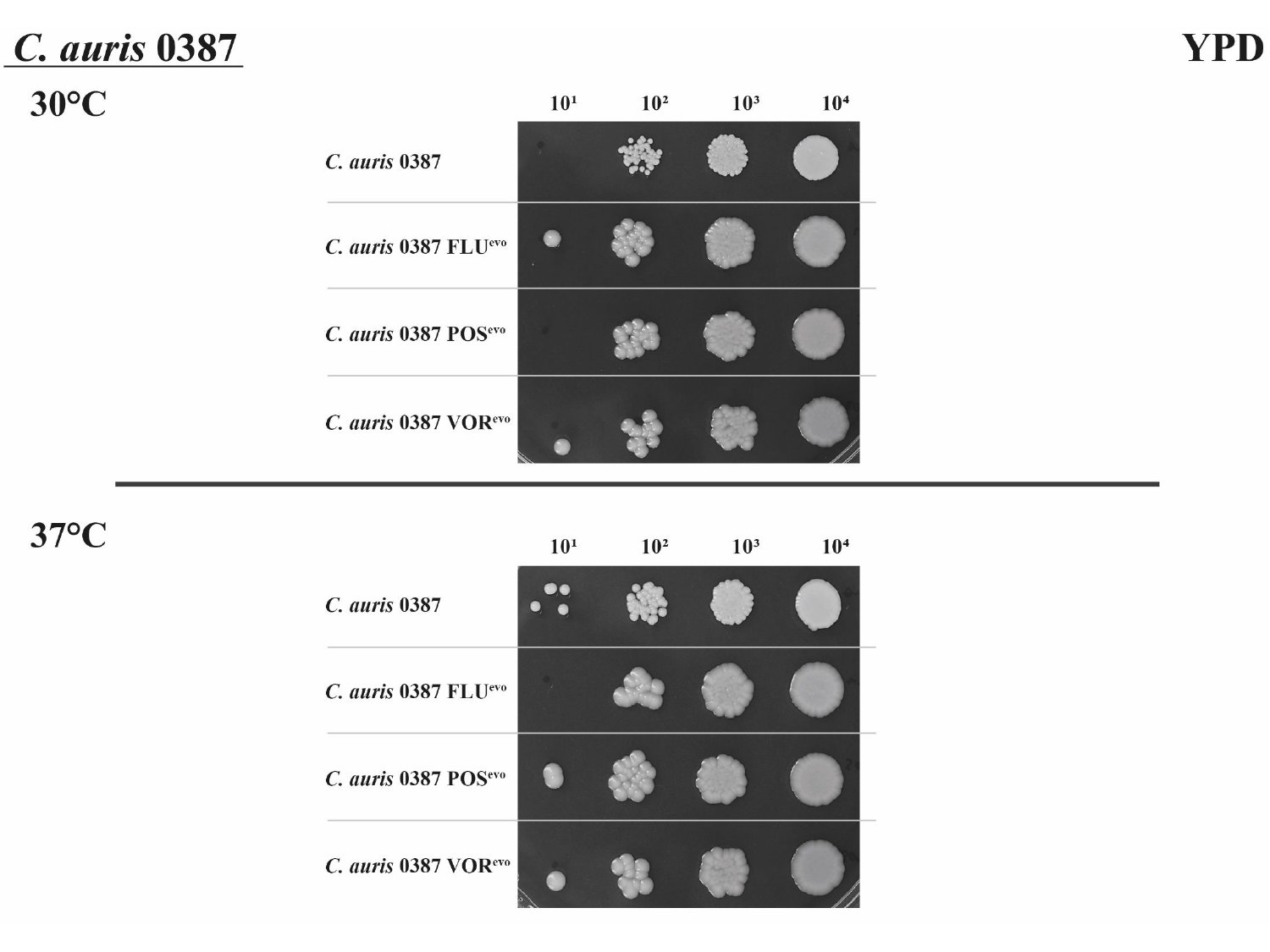

## Slide 14
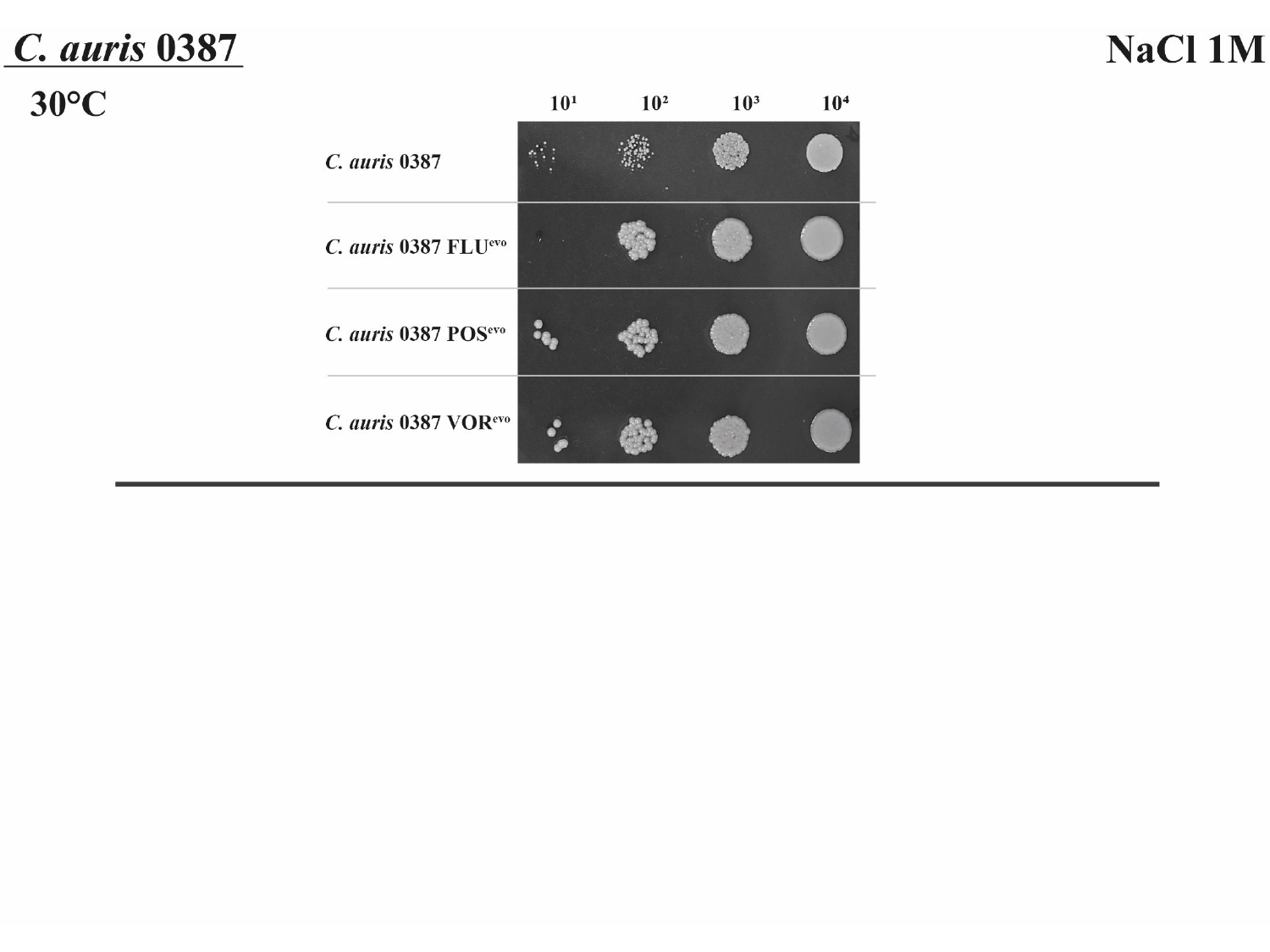

## Slide 15
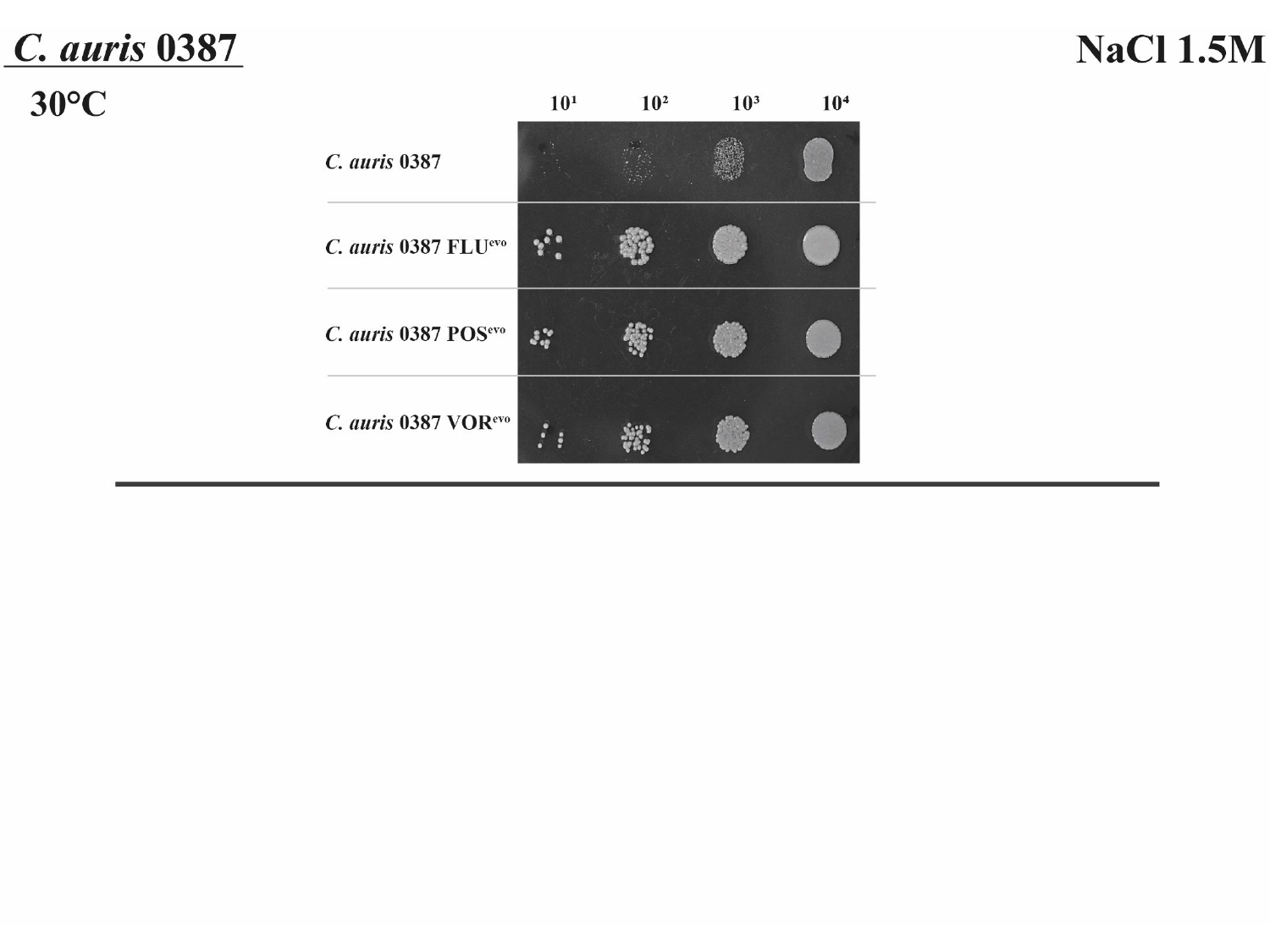

## Slide 16
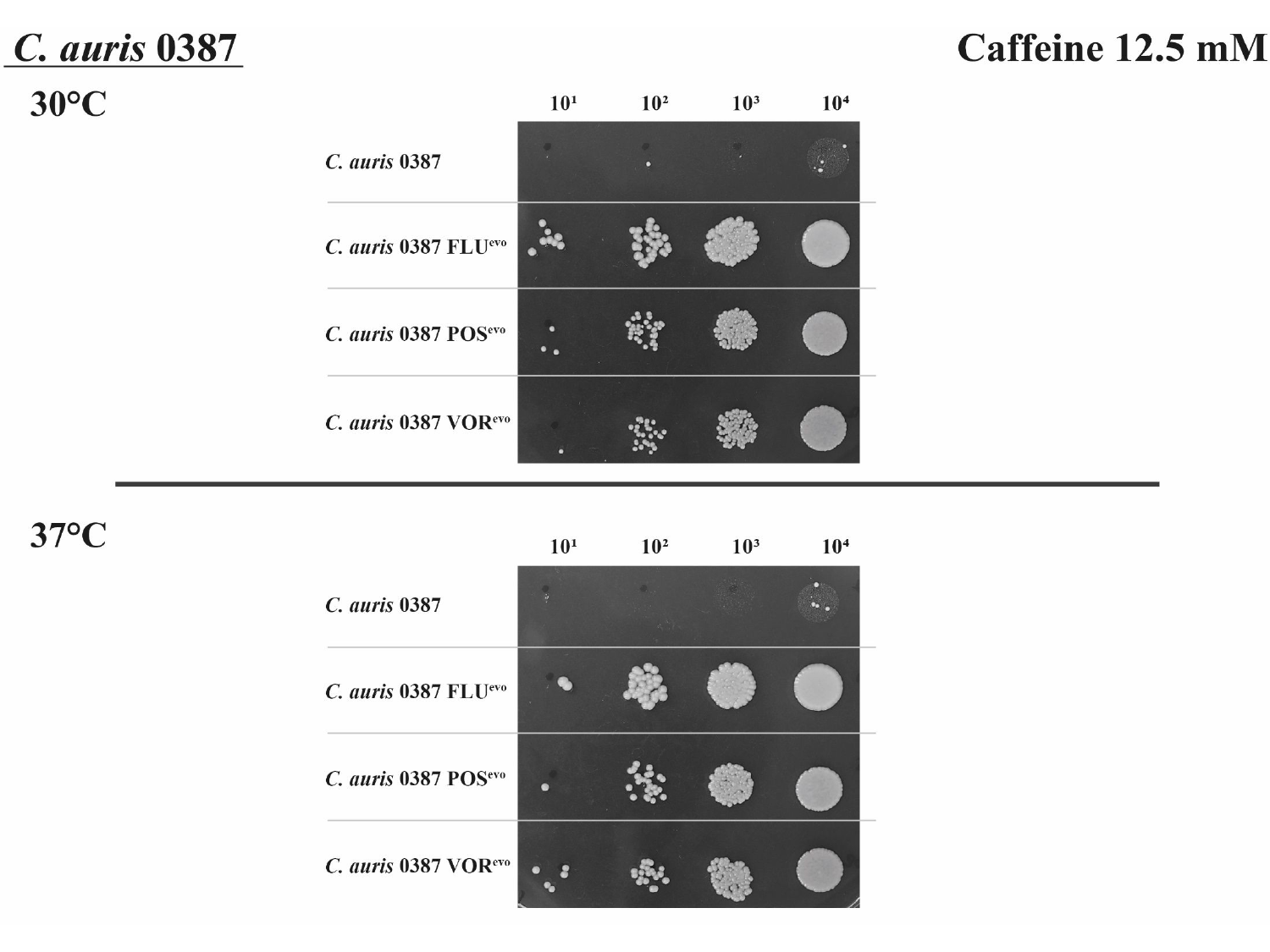

## Slide 17
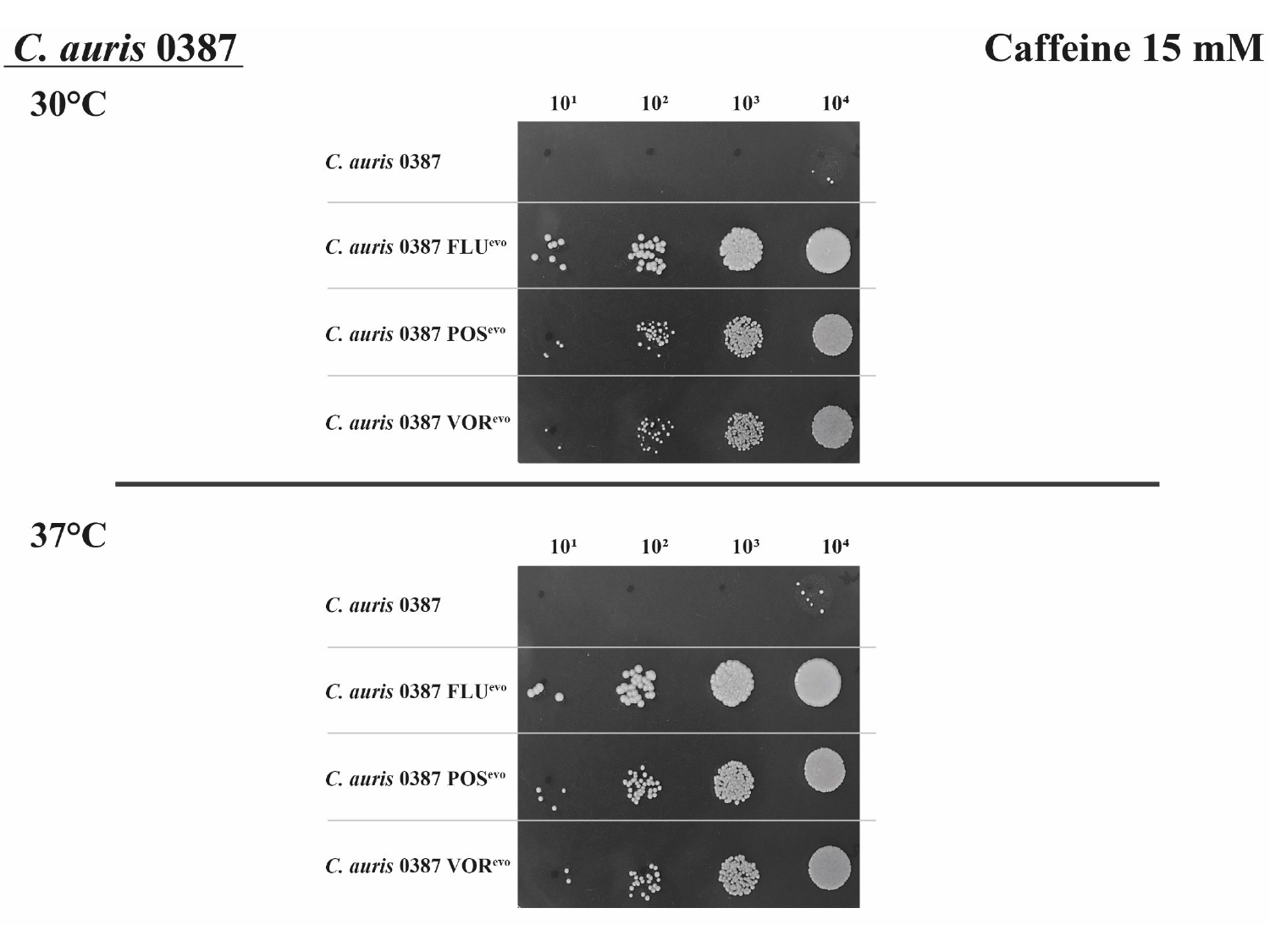

## Slide 18
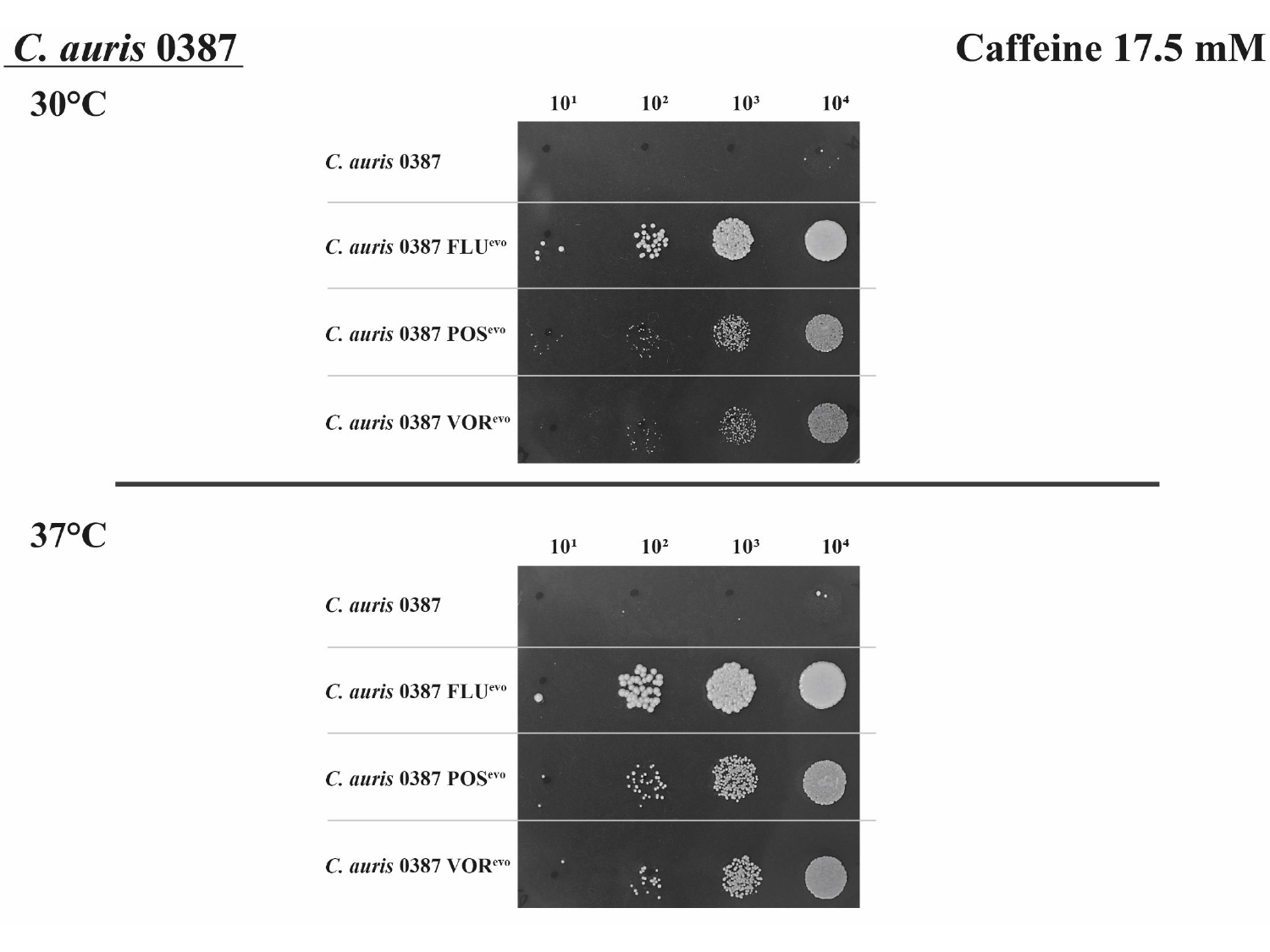

## Slide 19
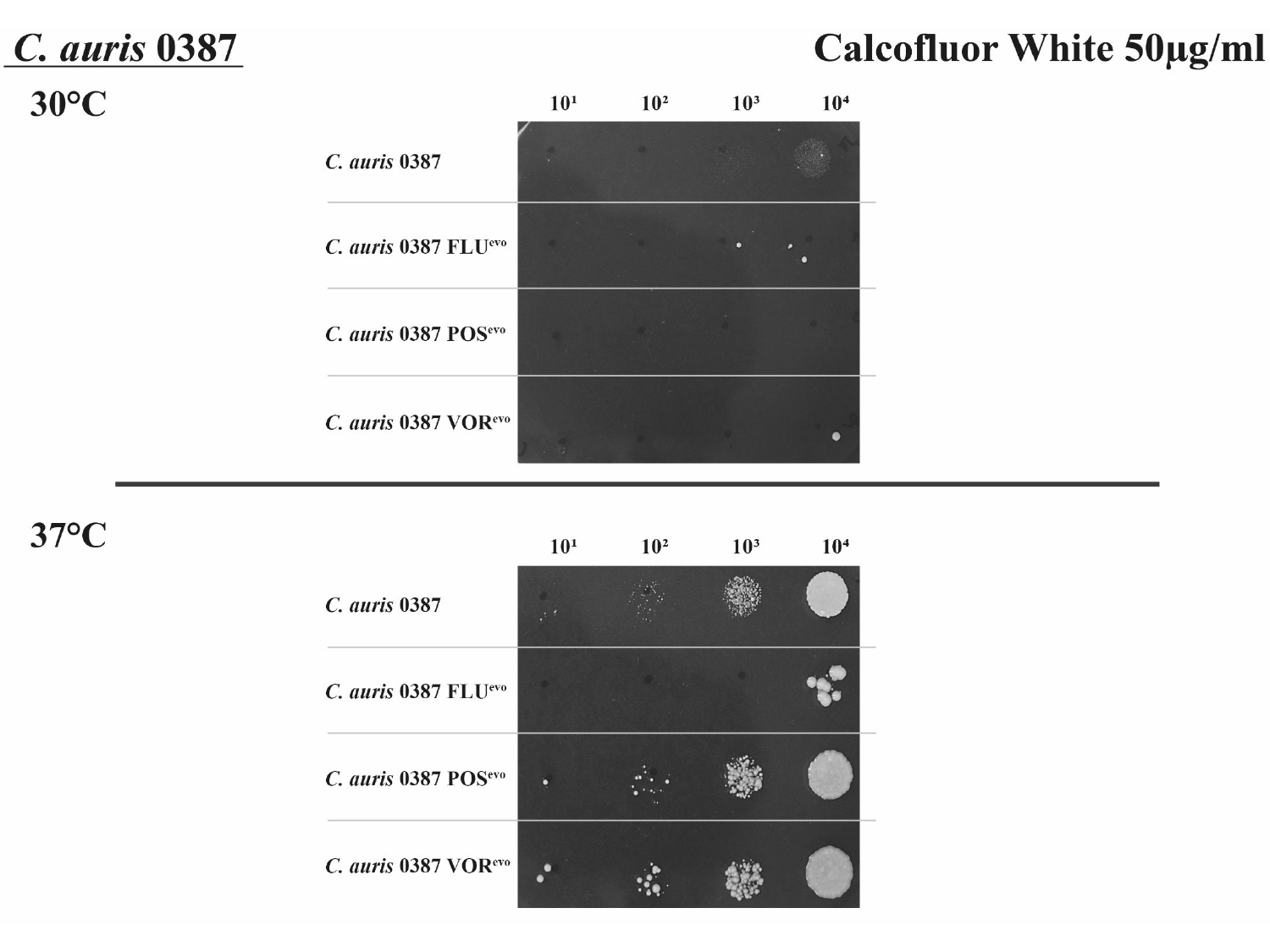

## Slide 20
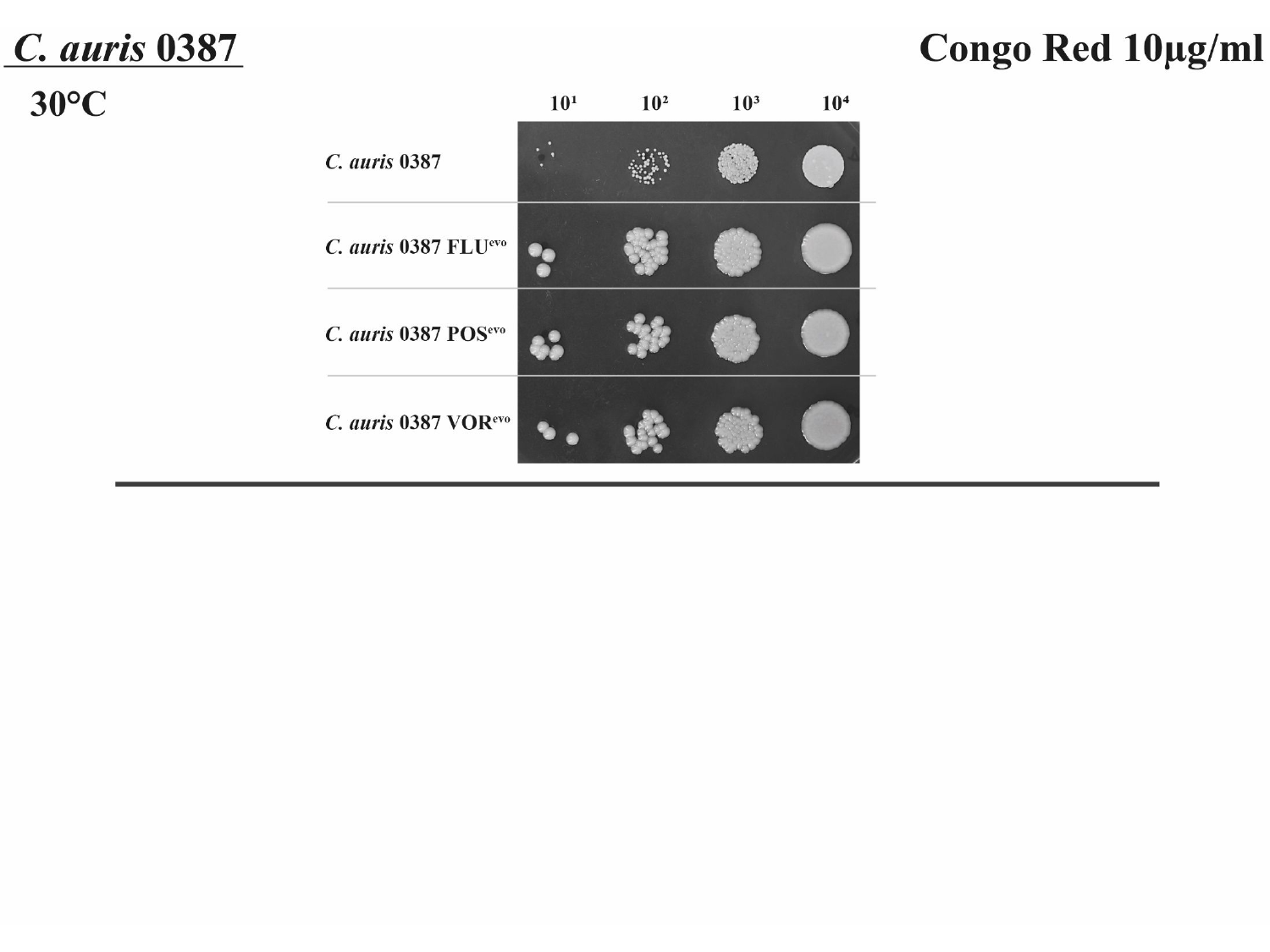

## Slide 21
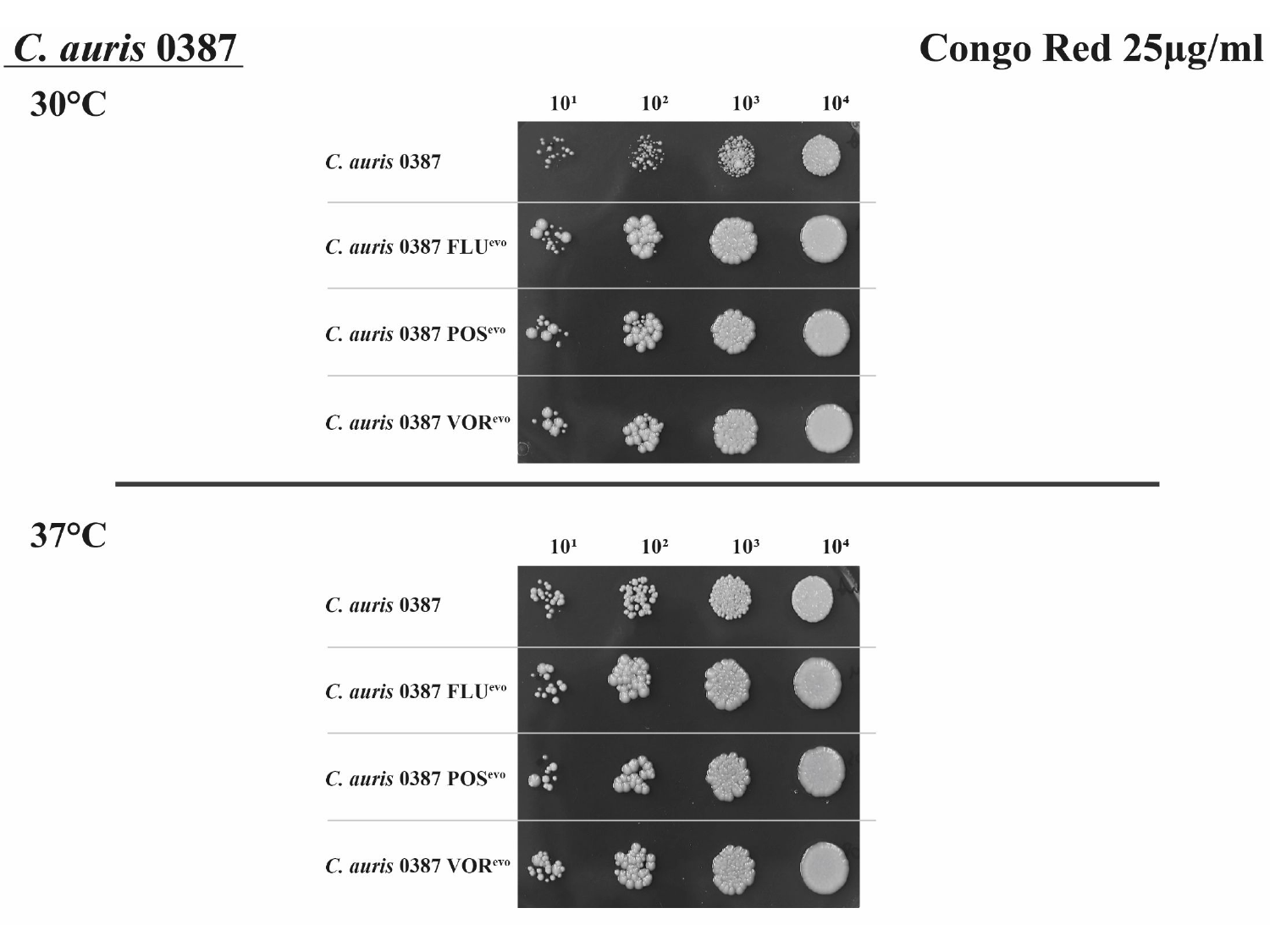

## Slide 22
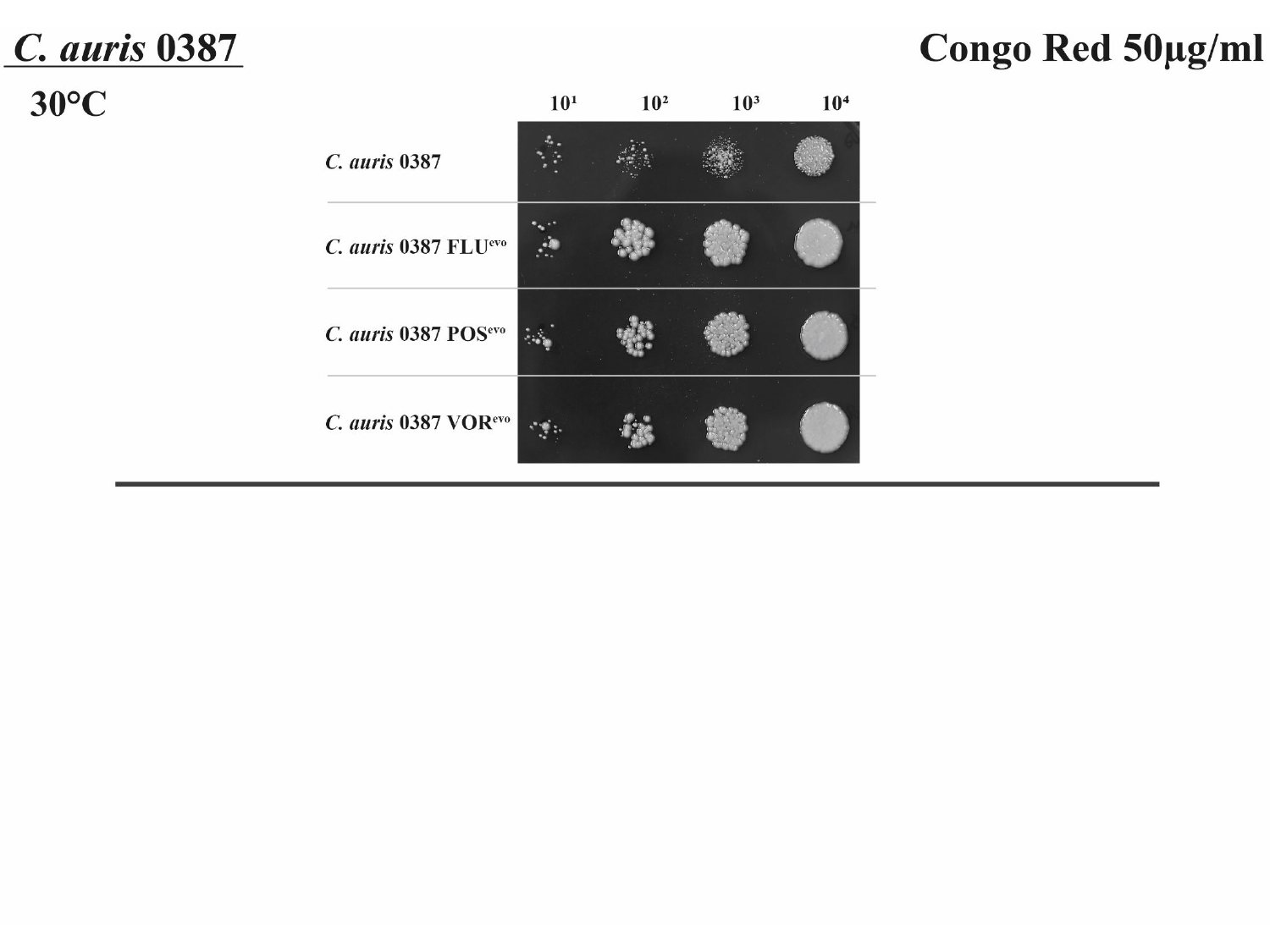

## Slide 23
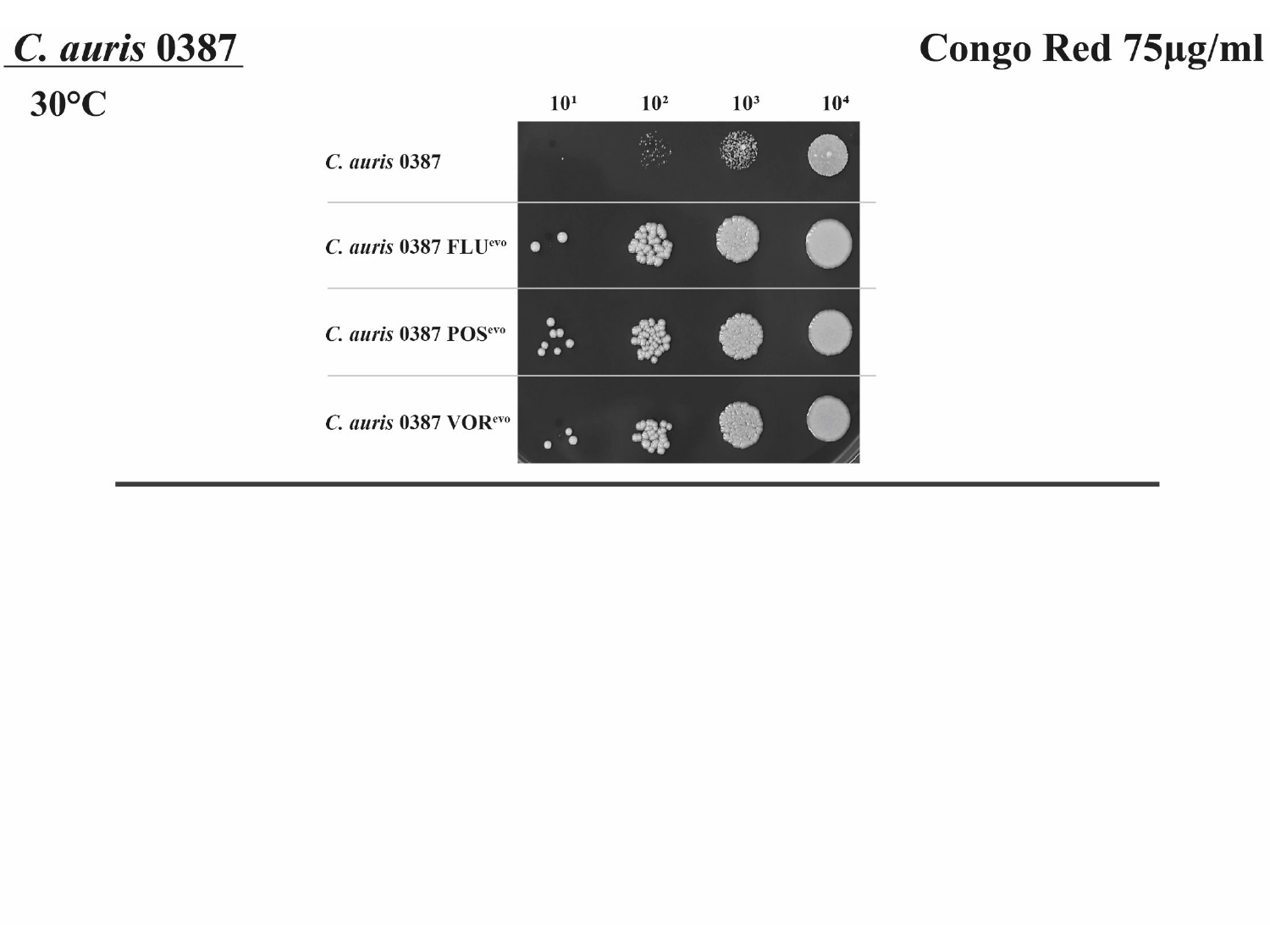

## Slide 24
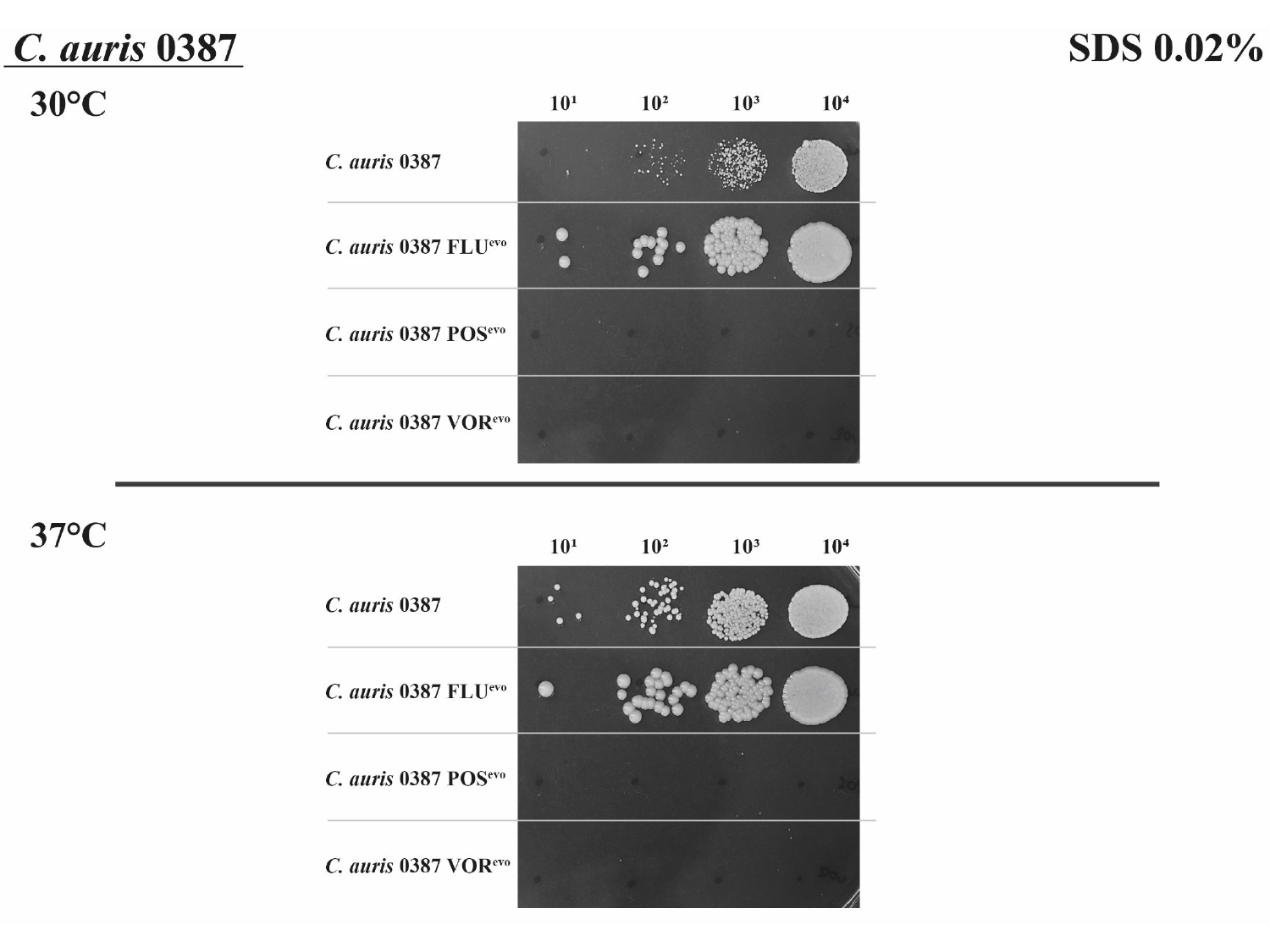

## Slide 25
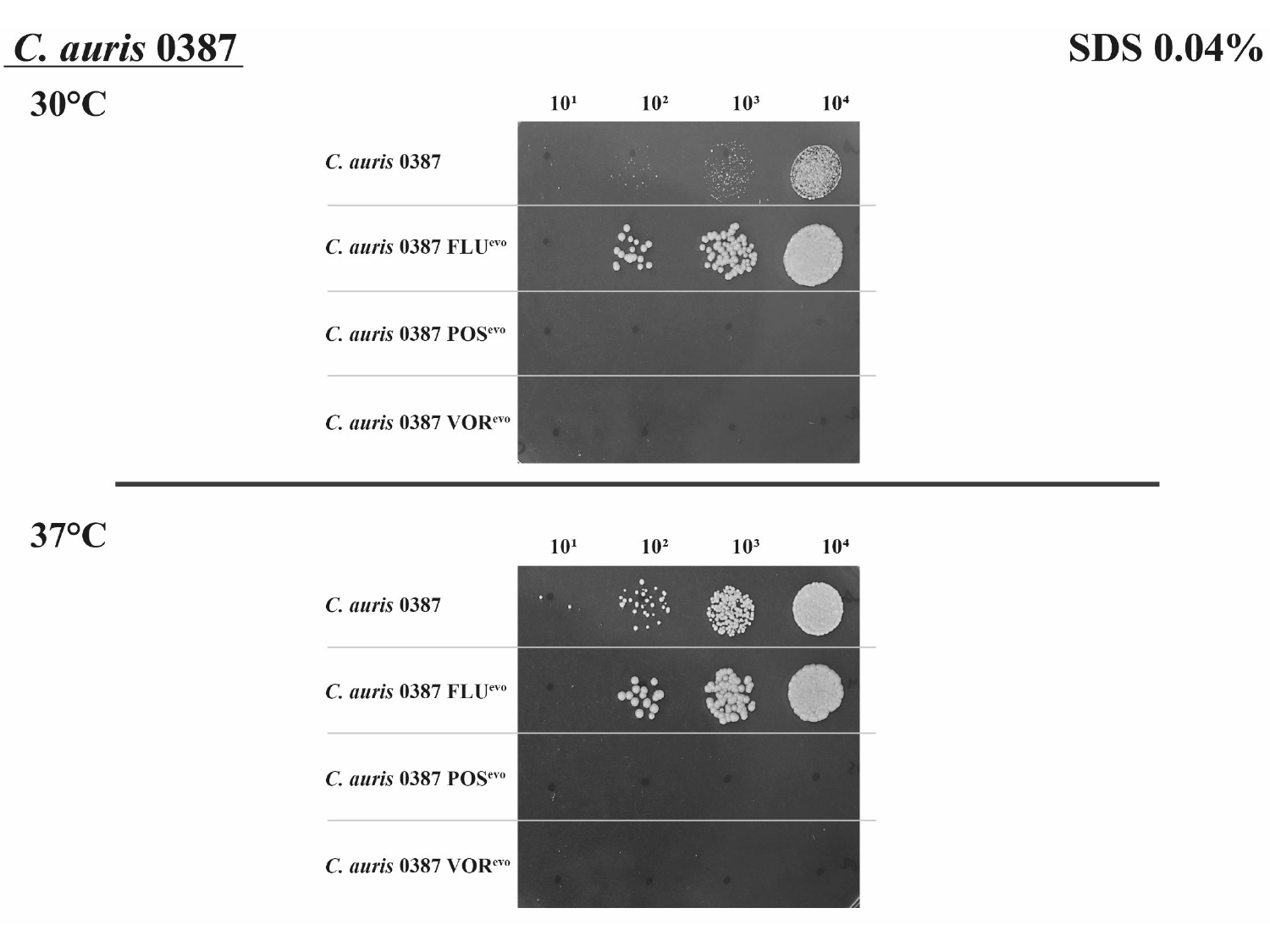

## Slide 26
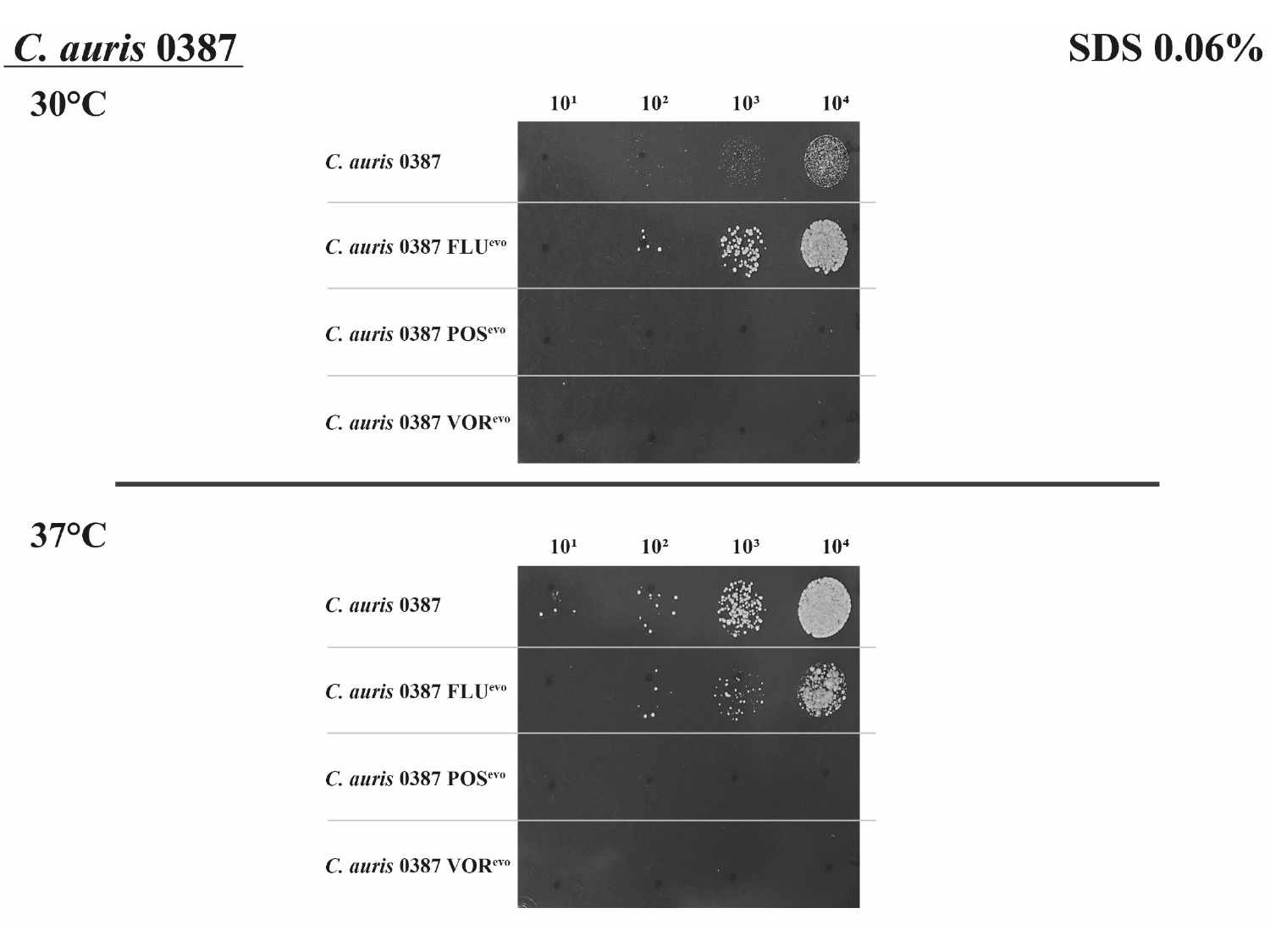

Supplement: Supplementary file 1 [file jof-09-01148-s001.zip › jof-2636826-Appendix S1.pptx]
