# Supplementary material for: Acquired Triazole Resistance Alters Pathogenicity-Associated Features in Candida auris in an Isolate-Dependent Manner
Source: J Fungi (Basel). 2023 Nov 28;9(12):1148. doi: 10.3390/jof9121148 (PMC10744493; doi:10.3390/jof9121148)
Supplement: Supplementary file 1 [file jof-09-01148-s001.zip › jof-2636826-supplementary tables and figures.pdf]

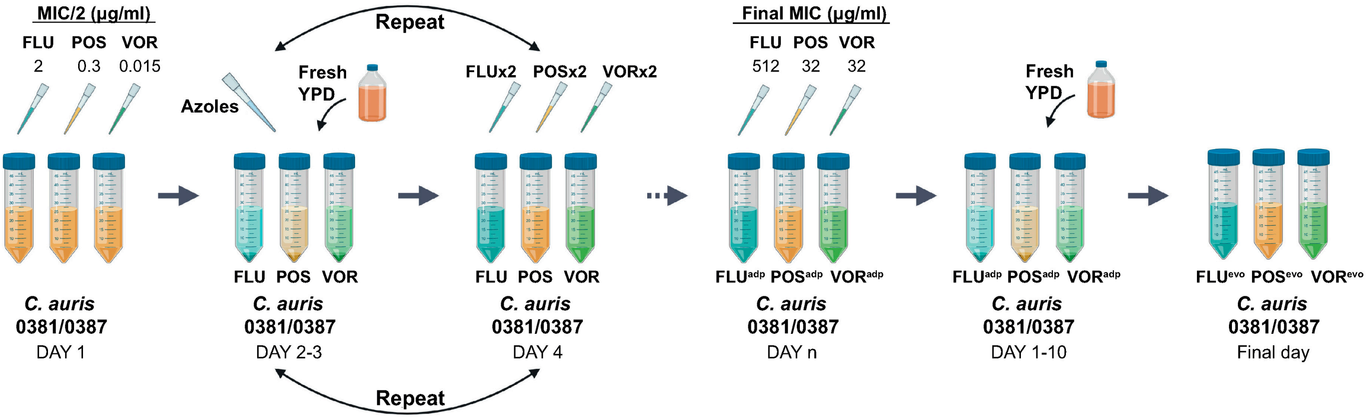

Figure S1. ***In vitro*** **microevolution process.**

Scheme of the *in vitro* microevolution process applied to generate the triazole resistant *C. auris* strains derived from the azole susceptible clinical isolates 0381 and 0387. FLU: fluconazole; POS: posaconazole; VOR: voriconazole.

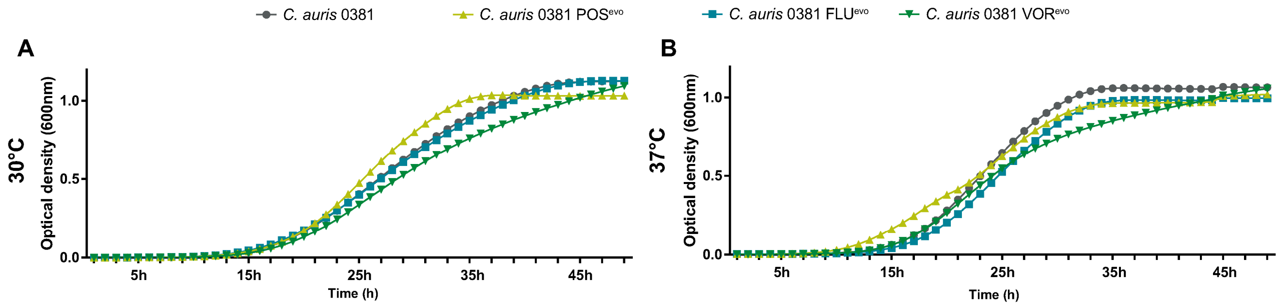

Figure S2. Growth kinetics of the generated strains compared to the parental isolates in complex media (YPD).

(A) Growth curve of the C. auris 0381 clinical isolate and the evolved strains at 30°C; **(B)** Growth curve of the C. auris 0381 clinical isolate and the evolved strains at 37°C. Growth curves represent the means of at least 30 data points from three independent experiments.

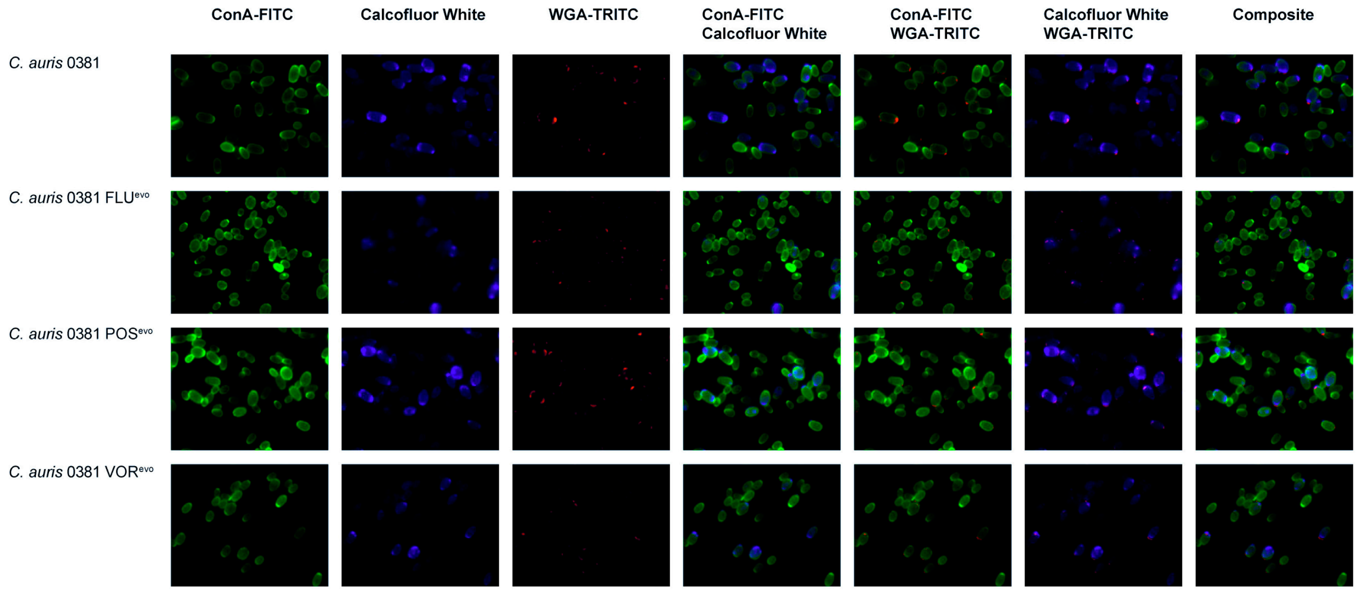

Figure S3. Fluorescence microscopic analysis of the cell-wall components of the 0381 isolate derived strains

Alpha-mannan content of the cell wall was determined by staining with ConA-FITC; chitin was stained by Calcofluor white, and chitin oligomer content was detected using WGA-TRITC. ConA: FITC conjugated Concavalin-A; WGA: TRITC conjugated Wheat Germ Agglutinin.

Table S1. Initial and final antifungal concentrations for the *in vitro* microevolution experiment

Antifungal drug concentrations used during the microevolution process. Initial concentrations for each antifungal were established as half of the MIC values determined for the clinical isolates. Final drug concentrations were chosen to confidently determine resistance.

Strains	Growth rate		Inflection point	
	30°C	37°C	30°C	37°C
**C. auris 0381**	0.218	0.355	25.3	19.8
**C. auris 0381 FLU^{evo}**	0.198	0.314	26.0	21.4
**C. auris 0381 POS^{evo}**	0.300	0.416	23.0	22.2
**C. auris 0381 VOR^{evo}**	0.198	0.254	27.5	27.2
**C. auris 0387**	0.220	0.508	31.2	21.0
**C. auris 0387 FLU^{evo}**	0.304	0.403	18.2	15.0
**C. auris 0387 POS^{evo}**	0.350	0.468	17.9	13.8
**C. auris 0387 VOR^{evo}**	0.371	0.558	17.8	13.9

Table S2. Growth curve analysis of the clinical isolates and evolved strains

Growing capacity of the evolved strains was monitored in complex media (YPD). Optical density (OD_{600nm}) values were registered, and the subsequent growth curves were analyzed in R environment, using 'Growthcurver' package.

m/z	**379,337**	**381,352**	**383,368**	**391,337**	**393,353**	**395,332**	**395,368**	**407,368**	**409,383**	**411,363**	**423,399**	**425,380**		
**tR (min)**	**6,1**	**6,46**	**6,78**	**5,48**	**4,24**	**6,4**	**6,27**	**4,55**	**6,83**	**4,29**	**7,06**	**4,54**		
**Sample**	**Ergosterole**	**Fecosterol/Episterol/Ergostadienol**	**Ergostaenol**	**?**	**?**	**?**	**14-Me-fecosterol**	**?**	**Lanosterol/Obtusifoliol**	**14-Me-ergostadien-diol**	**Eburicol**	**?**		
	$C_{28}H_{44}O$	$C_{28}H_{46}O$	$C_{28}H_{48}O$	$C_{29}H_{44}O$	$C_{29}H_{46}O$	$C_{28}H_{44}O_2$	$C_{29}H_{48}O$	$C_{30}H_{48}O$	$C_{30}H_{50}O$	$C_{29}H_{48}O_2$	$C_{31}H_{52}O$	$C_{30}H_{50}O_2$	**SUM**	
**1**	1,85E+10	3,21E+08	1,93E+07	0	0	0	0	1,37E+07	3,87E+08	0	0		1,93E+10	
**2**	1,96E+10	2,96E+08	1,74E+07	0	0	0	0	1,37E+07	4,07E+08	0	0		2,03E+10	**0381 wt**
**3**	1,77E+10	2,80E+08	1,23E+07	0	0	0	0	1,37E+07	3,84E+08	0	0		1,83E+10	
**4**	3,96E+09	2,01E+07	0	3,32E+07	9,74E+08	0	3,33E+09	1,19E+07	1,31E+10	1,71E+09	9,98E+08	1,79E+08	2,43E+10	
**5**	3,97E+09	2,02E+07	0	4,19E+07	1,88E+09	0	3,88E+09	6,79E+07	1,49E+10	3,43E+09	1,09E+09	2,30E+08	2,96E+10	**0381 wt FLU**
**6**	3,85E+09	2,07E+07	0	3,47E+07	1,14E+09	0	2,87E+09	4,65E+07	9,93E+09	2,07E+09	9,11E+08	1,90E+08	2,11E+10	
**7**	5,09E+09	2,26E+07	0	4,16E+07	9,71E+08	0	3,54E+09		1,25E+10	1,77E+09	8,20E+08	3,98E+08	2,51E+10	
**8**	4,57E+09	1,76E+07	0	3,70E+07	8,84E+08	0	3,99E+09		1,50E+10	1,59E+09	8,46E+08	4,53E+08	2,74E+10	**0381 wt POS**
**9**	4,40E+09	1,66E+07	0	3,09E+07	7,22E+08	0	3,95E+09		1,38E+10	1,28E+09	7,53E+08	3,67E+08	2,53E+10	
**10**	4,23E+09	8,67E+06	0	6,63E+07	7,75E+08	0	3,23E+09		1,28E+10	1,41E+09	1,11E+09	1,24E+08	2,37E+10	
**11**	4,50E+09	8,44E+06	0	9,59E+07	1,04E+09	0	3,01E+09		1,15E+10	1,86E+09	1,13E+09	1,53E+08	2,33E+10	**0381 wt VOR**
**12**	4,81E+09	1,16E+07	0	7,64E+07	9,29E+08	0	3,29E+09		1,18E+10	1,68E+09	1,21E+09	1,52E+08	2,40E+10	
**13**	1,78E+10	2,43E+08	3,92E+07	0	0	0	3,07E+07	1,17E+07	3,94E+08	0	0		1,86E+10	
**14**	1,75E+10	2,26E+08	3,48E+07	0	0	0	1,26E+07	1,34E+07	4,39E+08	0	0		1,82E+10	**0381 FLU**
**15**	1,90E+10	2,97E+08	3,81E+07	0	0	0	1,94E+07	1,22E+07	4,16E+08	0	0		1,98E+10	
**16**	1,02E+10	1,60E+08	3,65E+07	1,05E+08	5,10E+08	0	2,29E+09	5,79E+08	8,51E+09	1,46E+09	4,59E+08	1,04E+09	2,53E+10	
**17**	1,01E+10	1,33E+08	1,86E+07	1,10E+08	5,62E+08	0	2,23E+09	5,79E+08	7,94E+09	1,52E+09	4,30E+08	1,04E+09	2,46E+10	**0381 FLU FLU**
**18**	1,12E+10	1,28E+08	1,51E+07	1,29E+08	7,18E+08	0	2,56E+09	7,15E+08	9,14E+09	1,99E+09	4,78E+08	1,27E+09	2,84E+10	
**19**	3,93E+09	1,07E+10	2,75E+09	0	0	1,91E+08	0		1,36E+08	0	0		1,77E+10	
**20**	4,04E+09	1,12E+10	2,60E+09	0	0	1,96E+09	0		1,36E+08	0	0		2,00E+10	**0381 POS**
**21**	3,09E+09	7,42E+09	1,57E+09	0	0	1,42E+08	0		3,59E+08	0	0		1,26E+10	
**22**	2,92E+08	2,83E+08	1,85E+07	1,15E+07	8,99E+07	0	1,11E+10	1,73E+08	1,32E+10	2,18E+08	8,27E+08	1,05E+09	2,73E+10	
**23**	3,35E+08	3,07E+08	1,84E+07	1,24E+07	1,01E+08	0	1,25E+10	1,84E+08	1,34E+10	2,41E+08	9,07E+08	1,11E+09	2,91E+10	**0381 POS POS**
**24**	3,08E+08	3,15E+08	1,85E+07	1,37E+07	1,07E+08	0	1,28E+10	1,80E+08	1,31E+10	2,54E+08	8,56E+08	1,10E+09	2,90E+10	
**25**	1,25E+10	1,32E+08	1,51E+07	0	0	0	0		1,05E+08	0	0		1,28E+10	
**26**	1,32E+10	1,43E+08	1,82E+07	0	0	0	0		1,12E+08	0	0		1,35E+10	**0381 VOR**
**27**	1,40E+10	1,58E+08	1,79E+07	0	0	0	0		1,21E+08	0	0		1,43E+10	

**28**	4,01E+09	4,03E+08	3,35E+07	1,14E+08	8,36E+08	0	4,11E+09	4,17E+08	1,39E+10	1,76E+09	5,99E+08	1,08E+09	2,73E+10	
**29**	4,53E+09	4,15E+08	2,88E+07	1,24E+08	8,56E+08	0	4,41E+09	4,61E+08	1,57E+10	2,02E+09	6,58E+08	1,19E+09	3,04E+10	**0381 VOR VOR**
**30**	3,52E+09	3,24E+08	3,00E+07	9,90E+07	6,89E+08	0	3,35E+09	3,53E+08	1,20E+10	1,56E+09	4,95E+08	9,17E+08	2,34E+10	
**31**	1,36E+10	3,17E+08	1,12E+07	0	0	0	2,72E+06		2,59E+08	0	1,69E+07		1,42E+10	
**32**	1,62E+10	3,53E+08	1,33E+07	0	0	0	5,59E+06		2,89E+08	0	1,16E+07		1,69E+10	**0387 wt**
**33**	1,55E+10	3,26E+08	1,09E+07	0	0	0	5,15E+06		2,64E+08	0	1,07E+07		1,61E+10	
**34**	4,65E+09	3,14E+07	0	2,84E+07	1,11E+09	0	2,03E+09		5,46E+09	2,02E+09	9,92E+08	8,98E+07	1,64E+10	
**35**	4,28E+09	2,78E+07	0	2,58E+07	1,02E+09	0	1,98E+09		5,23E+09	1,86E+09	9,63E+08	8,68E+07	1,55E+10	**0387 wt FLU**
**36**	4,67E+09	2,89E+07	0	2,80E+07	1,13E+09	0	2,12E+09		5,63E+09	2,09E+09	1,03E+09	9,42E+07	1,68E+10	
**37**	4,35E+09	2,16E+07	0	2,88E+07	8,45E+08	0	2,14E+09		5,96E+09	1,58E+09	8,10E+08	7,96E+07	1,58E+10	
**38**	4,61E+09	2,49E+07	0	3,49E+07	1,02E+09	0	2,45E+09		6,33E+09	1,88E+09	8,86E+08	9,42E+07	1,73E+10	**0387 wt POS**
**39**	3,99E+09	2,19E+07	0	3,06E+07	9,87E+08	0	2,02E+09		5,69E+09	1,77E+09	7,85E+08	1,24E+08	1,54E+10	
**40**	4,58E+09	1,31E+07	0	4,94E+07	8,63E+08	0	1,96E+09		5,30E+09	1,58E+09	1,03E+09	6,23E+07	1,54E+10	
**41**	4,37E+09	1,66E+07	0	5,74E+07	8,64E+08	0	2,76E+09		6,74E+09	1,61E+09	9,25E+08		1,73E+10	**0387 wt VOR**
**42**	4,02E+09	1,50E+07	0	5,49E+07	9,81E+08	0	2,92E+09		7,21E+09	1,75E+09	1,01E+09		1,80E+10	
**43**	1,90E+10	3,51E+08	0	0	0	0	0		1,50E+08	0	0		1,95E+10	
**44**	1,84E+10	3,23E+08	0	0	0	0	0		1,48E+08	0	0		1,89E+10	**0387 FLU**
**45**	2,01E+10	3,32E+08	0	0	0	0	0		1,58E+08	0	0		2,06E+10	
**46**	5,31E+08	0	0	4,57E+07	1,60E+09	0	7,39E+09		1,85E+10	2,95E+09	1,51E+09	7,70E+08	3,33E+10	
**47**	4,77E+08	0	0	4,14E+07	1,35E+09	0	6,16E+09		1,59E+10	2,50E+09	1,45E+09	6,44E+08	2,85E+10	**0387 FLU FLU**
**48**	5,75E+08	0	0	4,65E+07	1,89E+09	0	7,64E+09		1,89E+10	3,45E+09	1,71E+09	8,03E+08	3,50E+10	
**49**	2,46E+07	1,19E+10	6,71E+08	0	0	5,04E+08	0		1,10E+08	0	0		1,33E+10	
**50**	2,96E+07	1,39E+10	7,03E+08	0	0	5,60E+08	0		1,37E+08	0	0		1,53E+10	**0387 POS**
**51**	3,16E+07	1,25E+10	8,15E+08	0	0	5,51E+08	0		1,34E+08	0	0		1,40E+10	
**52**	0	4,36E+08	1,17E+07	0	0	0	1,47E+10		1,27E+10	0	1,50E+09	5,45E+08	3,00E+10	
**53**	0	4,23E+08	6,76E+06	0	0	0	1,60E+10		1,24E+10	0	1,65E+09	5,46E+08	3,10E+10	**0387 POS POS**
**54**	0	3,90E+08	4,32E+06	0	0	0	1,37E+10		1,09E+10	0	1,63E+09	2,92E+08	2,70E+10	
**55**	2,43E+08	1,57E+10	7,17E+08	0	0	4,53E+08	2,27E+07		1,90E+08	0	1,66E+07		1,73E+10	
**56**	2,33E+08	1,28E+10	6,91E+08	0	0	4,04E+08	3,13E+06		1,11E+08	0	2,36E+06		1,42E+10	**0387 VOR**
**57**	0,00E+00	1,42E+10	1,04E+09	0	0	4,02E+08	4,31E+06		1,30E+08	0	3,66E+06		1,58E+10	
**58**	0	4,51E+08	3,64E+06	0	0	0	1,35E+10		1,18E+10	0	2,62E+09	1,52E+08	2,85E+10	
**59**	0	3,78E+08	2,70E+06	0	0	0	1,16E+10		1,01E+10	0	2,25E+09	1,25E+08	2,45E+10	**0387 VOR VOR**
**60**	0	4,45E+08	4,41E+06	0	0	0	1,39E+10		1,06E+10	0	2,37E+09	1,31E+08	2,75E+10	

Table S3. Orthologs of the selected efflux pump coding genes and primers used in this study

(A) List of primer sequences used for the RT-qPCR; (B) Orthologous genes of the efflux pumps selected for RT-qPCR based expression analysis

A

	Forward primer (5'-3')	Reverse primer (5'-3')
**ACT1**	GAAGGAGATCACTGCTTTAGCC	CTGTGTGGATTGGTGGCTC
**CDR1**	GGGCTGGAAGTGCAAGATTCC	CATCAAGCAAGTAGCCACCG
**SNQ2a**	GATGTTCAATCAGCTGCGGTG	GGCATCAAGCTCACAGGTTGA
**SNQ2b**	GGCAATTTCAACACGCCGCTG	GGGCGTCTCCTTCGAAGACC
**MDR1**	GAGAGACGAGCCCCAGCC	GGCACCCGCCATCATACTTC
**TPO3**	GCCAGACATCAACATGCCTCC	GAGAATCGGGCGTGTGAAGT

B

	**C. auris Systematic Name**	**S. cerevisiae ortholog**	**C. albicans ortholog**	**C. glabrata ortholog**
**ABC transporters**				
**CDR1**	B9J08_000164	PDR5	CDR1	PDH1
**CDR6**	B9J08_005430	YOLO75C	ROA1	CAGL0I08019g
**SNQ2a**	B9J08_001125	SNQ2	SNQ2 (orf19.5759)	SNQ2 (orf19.5759)
**SNQ2b**	B9J08_004452	SNQ2	SNQ2 (orf19.5759)	SNQ2 (CAGL0I04862g)
**MFS transporters**				
**MDR1**	B9J08_003981	FLR1	MDR1	FLR1
**TPO3**	B9J08_004775	TPO2	TPO3	TPO3

Table S4. LC-HRMS data of the sterol composition of the clinical isolates and evolved strains

Raw LC-HRMS data registered for the three technical parallels of each strain.

	C. auris 0381 (B11220)		C. auris 0387 (B8441)	
	Initial drug concentration (µg/ml)	Final drug concentration (µg/ml)	Initial drug concentration (µg/ml)	Final drug concentration (µg/ml)
**Fluconazole (FLU)**	2	512	2	512
**Posaconazole (POS)**	0.03	32	0.125	32
**Voriconazole (VOR)**	0.015	32	0.015	32

Table S5. Rapamycin MIC values

For the 0387 evolved strains MIC values of rapamycin were determined at 80% growth arrest after 24h, 36h and 48h at both 30°C and 37°C.

Strains	MIC (ng/ml)					
	24h		36h		48h	
	30°C	37°C	30°C	37°C	30°C	37°C
**C. auris 0387**	4,23	1,63	18,23	7,16	41,67	14,32
**C. auris 0387 FLU^{evo}**	187,50	250	>500	>500	>500	>500
**C. auris 0387 POS^{evo}**	36,46	15,63	52,08	20,83	62,50	31,25
**C. auris 0387 VOR^{evo}**	5,86	5,86	52,08	18,23	62,50	20,83
